# Validation and reliability of mechanical stiffness assessment tools in multilayered polyurethane phantom models of healthy and diabetic plantar soft tissues

**DOI:** 10.1038/s41598-025-21084-5

**Published:** 2025-09-30

**Authors:** Tülay Çevik Saldıran, Robert Schleip, Katja Bartsch, Wolfgang Bauermeister, Torsten Pohl, Thomas Horstmann

**Affiliations:** 1 Department of Physiotherapy and Rehabilitation, Faculty of Health Sciences, Bitlis Eren University, Bitlis, Turkey; 2 Conservative and Rehabilitative Orthopaedics, TUM School of Medicine and Health, Technical University of Munich, Campus D, Georg-Brauchle-Ring 60/62, 80992 Munich, Germany; 3 Department for Medical Professions, Diploma Hochschule, Bad Sooden-Allendorf, Germany; 4 Kharkiv National Medical University, Kharkiv, Ukraine

**Keywords:** Diabetic foot, Plantar soft tissue, Stiffness measurement, Phantom model, Foot biomechanics, Engineering, Health care, Medical research

## Abstract

Plantar soft tissue stiffness plays a crucial role in the development of diabetic foot complications, but in vivo assessments are constrained by anatomical variability and probe-induced measurement artifacts. To develop anatomically layered polyurethane phantom models mimicking healthy and diabetic plantar soft tissues and evaluate the reliability and concurrent validity of four mechanical stiffness assessment tools, [MyotonPRO, Shore Durometer, IndentoPRO, and Tissue Compliance Meter (TCM)] against Shear Wave Elastography (SWE). Six regional phantom models (calcaneus, midfoot, forefoot) with skin, fat pad, fascia, and muscle layers were fabricated. SWE was performed in no-contact mode to eliminate surface compression, improving measurement consistency. A total of 162 configurations were tested under blinded and randomized conditions. Intra- and inter-rater reliability was assessed using ICCs; concurrent validity was evaluated via correlation and regression analyses with SWE. All devices demonstrated excellent reliability (ICC range: 0.88–0.99). SWE-derived stiffness was significantly higher in diabetic models, especially in the calcaneal and midfoot regions (*p* < 0.001). IndentoPRO showed the highest correlation with SWE (r = 0.91), followed by MyotonPRO (r = 0.87), TCM (r = 0.85), and Durometer (r = 0.78). TCM exhibited the highest predictive value (R^2^ = 0.502) and most consistent performance across diabetic regions. The developed phantom models offer a standardized platform for evaluating stiffness assessment tools. While SWE remains the reference standard, mechanical devices, particularly TCM and IndentoPRO, demonstrated valid and reproducible performance. This phantom-based approach holds promise for supporting medical device development, regulatory validation, and preclinical testing in diabetic foot biomechanics.

**Supplementary Information:**

The online version contains supplementary material available at 10.1038/s41598-025-21084-5.

## Introduction

Diabetes mellitus is a pervasive and escalating global health challenge, currently affecting approximately 589 million adults aged 20–79 years, corresponding to 11.1% of the global adult population^1^. This number is projected to exceed 850 million by 2050, underscoring the urgent need for effective prevention and management strategies^2^. Among its numerous systemic complications, biomechanical alterations in plantar soft tissues are of particular clinical relevance due to their essential roles in weight-bearing, locomotion, and the prevention of foot ulceration^3–6^. These alterations are primarily driven by the pathological accumulation of advanced glycation end products (AGEs)^7,8^, which induce aberrant collagen cross-linking, reduced microvascular perfusion, and extracellular matrix remodeling, including changes in elastin fibers and glycosaminoglycans^4,6,9^. Collectively, these changes lead to increased tissue stiffness, impaired load distribution, and heightened susceptibility to pressure-related injuries^3,10^. This biomechanical deterioration is further exacerbated by peripheral neuropathy, which compromises protective sensation and significantly contributes to the development of diabetic foot ulcers, a major source of morbidity and healthcare burden^11–13^.

The human plantar surface comprises multilayered soft tissues, including skin, the heel fat pad, the plantar fascia, and the intrinsic foot muscles (IFMs). Each of these structures plays a distinct role in shock absorption, balance, and propulsion^14,15^. In individuals with diabetes, however, these tissues frequently undergo region-specific degeneration. Reduced skin elasticity and thinning are commonly observed, particularly in high-pressure regions such as the metatarsal heads and heel^6,12,16^. The fat pad may become fragmented and fibrotic, reducing its cushioning capacity^17^, while the plantar fascia often thickens and loses compliance^18,19^. Furthermore, IFMs, notably the abductor hallucis and flexor digitorum brevis, are prone to atrophy and fatty infiltration, leading to compromised arch support and postural control^16,20–25^. These cumulative structural changes disrupt plantar pressure distribution, contribute to gait abnormalities, and increase the risk of ulceration^26^.

Accurate and reliable assessment of plantar soft tissue stiffness is essential to improve the understanding and management of diabetes-related complications^12,13,19,27–29^. However, current in vivo techniques are limited by anatomical variability, restricted access to deeper layers, and operator-dependent inconsistencies, which reduce reproducibility and comparability across studies^30^. These challenges are particularly evident in anatomically complex regions such as the midfoot, where achieving consistent and reproducible in vivo measurements remains difficult^31^. Phantom models offer a robust solution by eliminating structural variability, enabling controlled replication of pathological changes such as diabetes-related stiffening, and providing standardized testing independent of operator expertise^32^. This advantage is especially relevant for midfoot assessment, where phantoms allow consistent and objective measurements under controlled conditions^32–37^. Unlike direct in vivo testing, which is confounded by inter-individual variability and clinical heterogeneity, phantom-based approaches ensure reproducibility, controllability, and reliability. They also remove patient-related risks and ethical concerns, thereby strengthening their role in preclinical research, device development, regulatory calibration, and educational applications^34,38,39^. Multilayer phantom models, in particular, enable reproducible simulation of pathological conditions such as increased stiffness in diabetes, supporting comparative device evaluation under standardized conditions. Although polyurethane-based phantoms are widely employed in imaging calibration and ultrasound validation studies^32,40^, their application to musculoskeletal and diabetic foot research remains limited. Importantly, polyurethane gels can be tuned to mimic the viscoelastic properties of soft tissues and layered to reflect the anatomical complexity of the plantar region^41^.

Several devices are available for assessing soft tissue stiffness, each employing distinct operational principles and penetrating to varying tissue depths. Shear Wave Elastography (SWE) is a non-invasive, ultrasound-based imaging modality that quantifies tissue elasticity by measuring shear wave velocity, and has emerged as a reference method in musculoskeletal research due to its ability to provide reproducible, depth-specific stiffness estimates without operator-induced compression^42^. In contrast, mechanical tools such as the Shore Durometer^31^, MyotonPRO^43^, IndentoPRO^44^, and Tissue Compliance Meter (TCM)^45^ offer portable and cost-effective alternatives for biomechanical evaluation. While these tools have demonstrated utility in both clinical^31,43–46^ and experimental settings^35^, there is a lack of comparative data under standardized conditions, particularly in diabetic-specific plantar models.

To address these challenges, the primary aim of this study was to construct anatomically accurate, multilayered polyurethane phantom models that replicate healthy and diabetic plantar soft tissues, incorporating region-specific configurations for the heel, midfoot, and forefoot. These phantoms, designed to replicate the layered structure of skin, fat pad, fascia, and intrinsic muscles, served as standardized platforms to evaluate the reliability and construct validity of four mechanical stiffness assessment tools (MyotonPRO, Shore Durometer, IndentoPRO, and TCM), using SWE as the reference standard. We hypothesized that the stiffness measurements obtained from each mechanical device would be significantly correlated with SWE-derived values, indicating their concurrent validity, and that all devices would demonstrate high intra- and inter-rater reliability under blinded, standardized conditions.

## Materials and methods

### Study design and setting

This experimental validation study was conducted between January and June 2025 at a university in Germany (institution blinded for review). As the study involved only synthetic models, ethical approval was not required.

### Phantom model development

Phantom models were constructed to represent three anatomical regions of the plantar foot: calcaneal (heel), midfoot, and forefoot. Each region was modeled in both healthy and diabetic versions, resulting in six distinct phantom configurations. The layer structures were designed based on detailed anatomical literature^47–49^. Specifically, the heel region included skin, fat pad, and plantar fascia; the midfoot region included skin, fat pad, fascia, and the flexor digitorum brevis muscle; and the forefoot region included skin, fat pad, fascia, and the abductor hallucis muscle.

Target stiffness values for each anatomical layer were determined based on a comprehensive review of published in vivo elastography and cadaveric indentation studies, ensuring physiological relevance of the mechanical properties simulated (see Supplemental File 1). This allowed each phantom model to replicate region-specific stiffness characteristics observed in both healthy and diabetic populations. Technogel® was selected as the primary construction material due to its favorable viscoelastic behavior, biocompatibility, and established use in musculoskeletal phantom development. Compared to other elastomers such as silicone or agar-based gels, Technogel offers superior layering stability, consistent surface compliance, and customizable stiffness ranges within the Shore-OOO scale—traits critical for simulating the nuanced mechanical alterations associated with diabetic foot pathology under standardized experimental conditions.

### Material fabrication and stiffness calibration

**Fig. 1:**
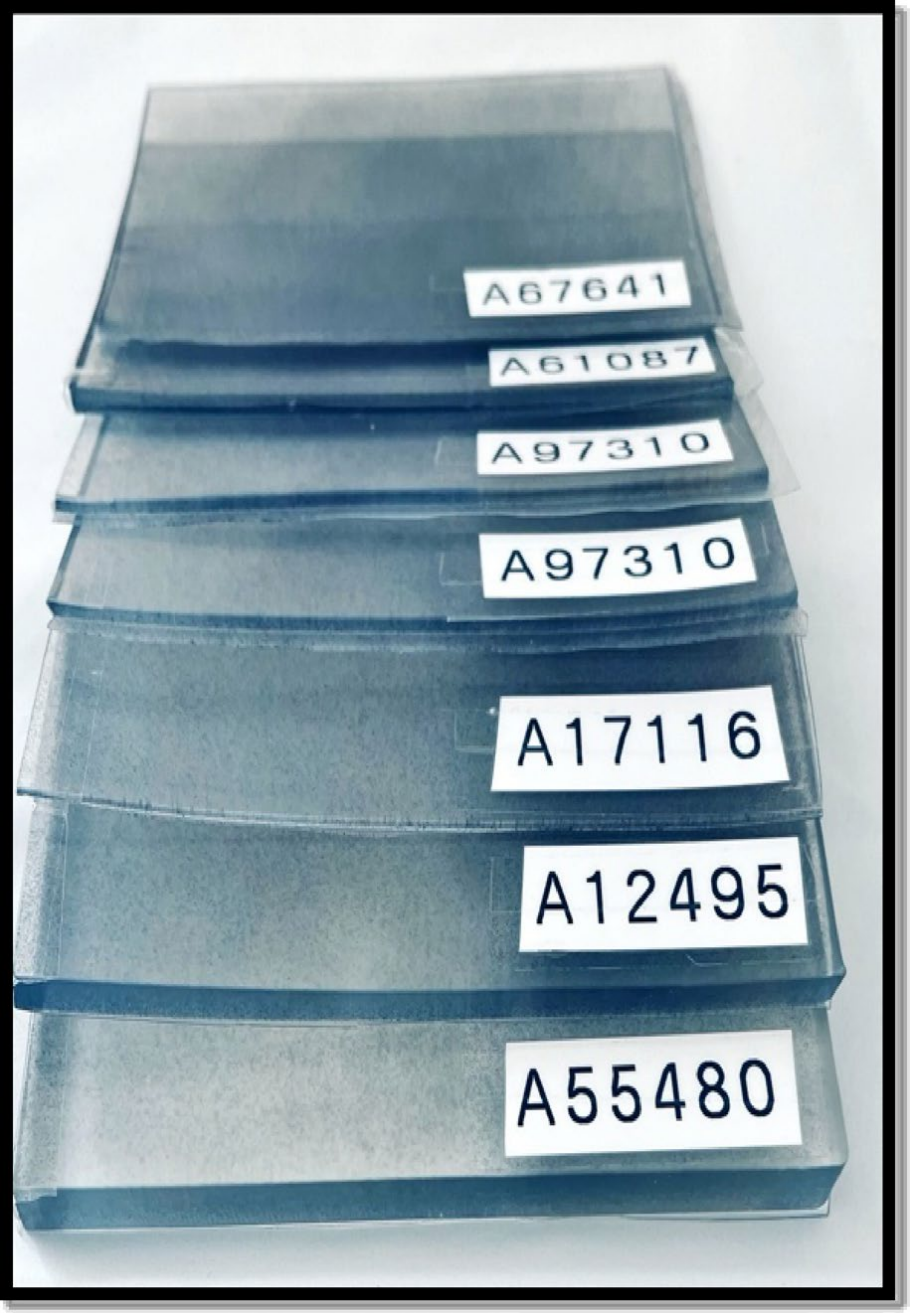
Polyurethane gel pads ınfused with micro-silica particles.

Each anatomical layer was fabricated using medical-grade polyurethane gel (Technogel GmbH, Germany), mixed with micro-silica particles to enhance both mechanical stability and ultrasound signal transmission. Layers were cast in standardized molds (15 × 10 cm) and sealed with a 25 μm elastic membrane to preserve hydration and surface characteristics during measurement (Fig. 1).

Initial mechanical calibration relied on Shore-OOO hardness ratings provided by the manufacturer (e.g., Shore-OOO 56), determined according to ASTM D2240, the standardized protocol issued by the American Society for Testing and Materials. Shore-OOO was specifically chosen over other durometer scales (e.g., Shore-A, Shore-OO) due to its higher sensitivity in the low-modulus range, which is most relevant for simulating soft biological tissues. Its fine resolution enables better discrimination of subtle stiffness differences among soft tissue analogs, particularly in diabetic models where small mechanical variations are clinically meaningful. Shore-OOO values were converted into estimates of Young’s modulus (*E*, in kPa) using established mathematical modeling methods^50^. In parallel, to ensure compatibility with elastographic measurements, *E* was also derived from shear wave velocity (*V*, in m/s) using the standard equation:

1 $$E = 3 \cdot p \cdot V^{2}$$

where ρ is the assumed tissue density (1000 kg/m^3^)^30^. This dual-calibration approach ensured consistency between mechanical and elastographic stiffness interpretations^30,51^. Final mechanical properties across all phantom configurations are summarized in Table 1.

**Table 1:** Mechanical properties by layer and region: shore-OOO, Young’s modulus, and shear wave velocity.

Healthy layers: calcaneal region				Healthy layers: midfoot region				Healthy layers: forefoot region			
Layer	Shore-OOO	E (kPa)	SWV (m/s)	Layer	Shore-OOO	E (kPa)	SWE (m/s)	Layer	Shore-OOO	E (kPa)	SWE (m/s)
A48742	20	33.9	3.4	A37718	35	60.1	4.6	A11108	45	89.7	5.5
B66583	35	59.1	4.8	A45327	40	73.8	5.4	A39415	20	35.2	3.4
A46490	55	135.3	6.9	A98575	45	88.5	5.4	A15481	30	50.9	4.1
A21083	20	34.9	3.4	B90057	20	33.3	3.4	A91825	40	71.2	4.9
A34907	35	61.1	4.6	B30216	25	41.4	3.8	A46742	15	29.7	3.2
A88020	50	129.6	6.5	B22332	35	61.9	4.5	A26181	25	41.0	3.7
A11179	35	58.5	4.8	B32372	35	61.0	4.5	A36718	35	61.4	4.5
B54659	45	89.6	5.6	B54659	45	91.4	5.5	A21917	15	27.9	3.1
B64504	50	109.9	6.3	B64504	50	106.3	5.6	A21083	20	35.6	3.4
				B72788	10	24.4	2.9	A86691	30	52.5	4.2

Diabetic layers: calcaneal region				Diabetic layers: midfoot region				Diabetic layers: forefoot region			
Layer	Shore-OOO	E (kPa)	SWV (m/s)	Layer	Shore-OOO	E (kPa)	SWV (m/s)	Layer	Shore-OOO	E (kPa)	SWV (m/s)
A66578	30	49.2	4.1	B10465	50	109.9	6.5	A96785	50	106.9	6.0
A62934	45	91.3	5.5	B42029	55	138.9	6.8	A94024	35	59.4	4.5
A19599	65	220.8	8.6	B77153	60	173.6	7.6	A90361	45	91.5	5.8
A35907	30	50.8	4.1	B37649	30	51.4	4.1	A73645	60	177.0	7.7
A90275	40	73.5	4.9	B17501	35	63.9	4.6	A93544	30	50.2	4.1
A88020	50	115.1	6.2	B35465	40	72.3	4.9	B66583	35	62.2	4.6
A62934	45	87.8	5.7	A48453	25	41.4	3.8	A98575	45	86.1	5.7
A97785	50	107.2	5.9	A13937	35	64.2	4.7	B72788	17	31.1	3.2
A67641	55	128.8	6.6	A31618	40	76.9	5.1	A86691	25	43.4	3.8
				A67931	20	33.3	3.7	A36907	30	47.9	4.0

Shore-OOO = Shore hardness scale (OOO range); E (kPa) = Elastic modulus in kilopascals; SWV (m/s) = Shear wave velocity; msec = Milliseconds.

### Layer variability and validation

Thickness and stiffness parameters for each tissue layer (skin, fat pad, fascia, and intrinsic muscle) were selected based on an extensive review of in vivo and cadaveric studies, as detailed in Supplemental File 1. For both healthy and diabetic models, each tissue type was constructed in three mechanical variants—representing minimum, average, and maximum stiffness values reported in the literature^35,52^. Accordingly, the heel region included 18 layers (9 healthy, 9 diabetic), while the midfoot and forefoot each comprised 20 layers (10 healthy, 10 diabetic).

All layers were evaluated using shear wave elastography (SWE) by two independent raters. Only those conforming to the literature-based target stiffness ranges were incorporated into the final multilayer assemblies. Each validated layer was assigned a coded label to ensure assessor blinding. Ultimately, six complete multilayer phantom models (three anatomical regions × two health conditions) were constructed. A total of 162 unique model configurations were generated by systematically altering one layer at a time. The full coding system and matrix of layer combinations are provided in Supplemental File 2.

### Measurement procedures

All measurements were conducted on a rigid support surface under standardized laboratory conditions (22 ± 1 °C; 50% relative humidity) to minimize potential base interference. Assessors were blinded to both the phantom type and anatomical region to reduce bias.

For each device and anatomical layer, three consecutive measurements were obtained. Values with a coefficient of variation (CV) greater than 5% were excluded to ensure measurement reliability. The remaining two or three valid values were averaged to calculate the final stiffness value for that layer. This exclusion criterion and averaging procedure were applied uniformly across all devices and phantom configurations to maintain consistency and transparency (Table 2).

**Table 2:** List of phantom layers designed for the calcaneal, midfoot, and forefoot regions in healthy and diabetic models.

Model region	Condition	Thickness (mm)	Shore-OOO	Stiffness variations		
				V1	V2	V3
*Calcaneal model*						
Plantar skin	Healthy	1.6*	20–56	20	35	56
Plantar fat pad	Healthy	16	20–52	20	35	50
Plantar fascia	Healthy	3	35–47	35	45	50
*Calcaneal model*						
Plantar skin	Diabetic	2	30–62+	30	45	65
Plantar fat pad	Diabetic	16	29–52	30	40	50
Plantar fascia	Diabetic	4	43–53	45	50	55
*Midfoot model*						
Plantar skin	Healthy	0.75 *	39–43	35	40	45
Plantar fat pad	Healthy	7	24–28	20	25	30
Plantar fascia	Healthy	2	34–47	35	45	50
Intrinsic foot muscle	Healthy	10	3–13	10		
*Midfoot model*						
Plantar skin	Diabetic	0.6*	49–51	50	55	60
Plantar fat pad	Diabetic	4	30–41	30	35	40
Plantar fascia	Diabetic	3	25–38	25	35	40
Intrinsic foot muscle	Diabetic	9	> 13	20		
*Forefoot model*						
Plantar skin	Healthy	2.5	44		45	
Plantar fat pad	Healthy	7	23–40	20	30	40
Plantar fascia	Healthy	1	15–34	15	25	35
Intrinsic foot muscle	Healthy	11	6–30	15	20	30
*Forefoot model*						
Plantar skin	Diabetic	1.8	51		51	
Plantar fat pad	Diabetic	8	37–60	35	45	60
Plantar fascia	Diabetic	1	28–46	30	35	45
Intrinsic foot muscle	Diabetic	11	17–27	17	25	30

*Due to technical constraints, 1 mm gel pads were chosen over the 1.6 mm, 0.75 mm, and 0.6 mm alternatives, as production below 1 mm thickness was not feasible.

#### Shear wave elastography (SWE)

**Fig. 2:**
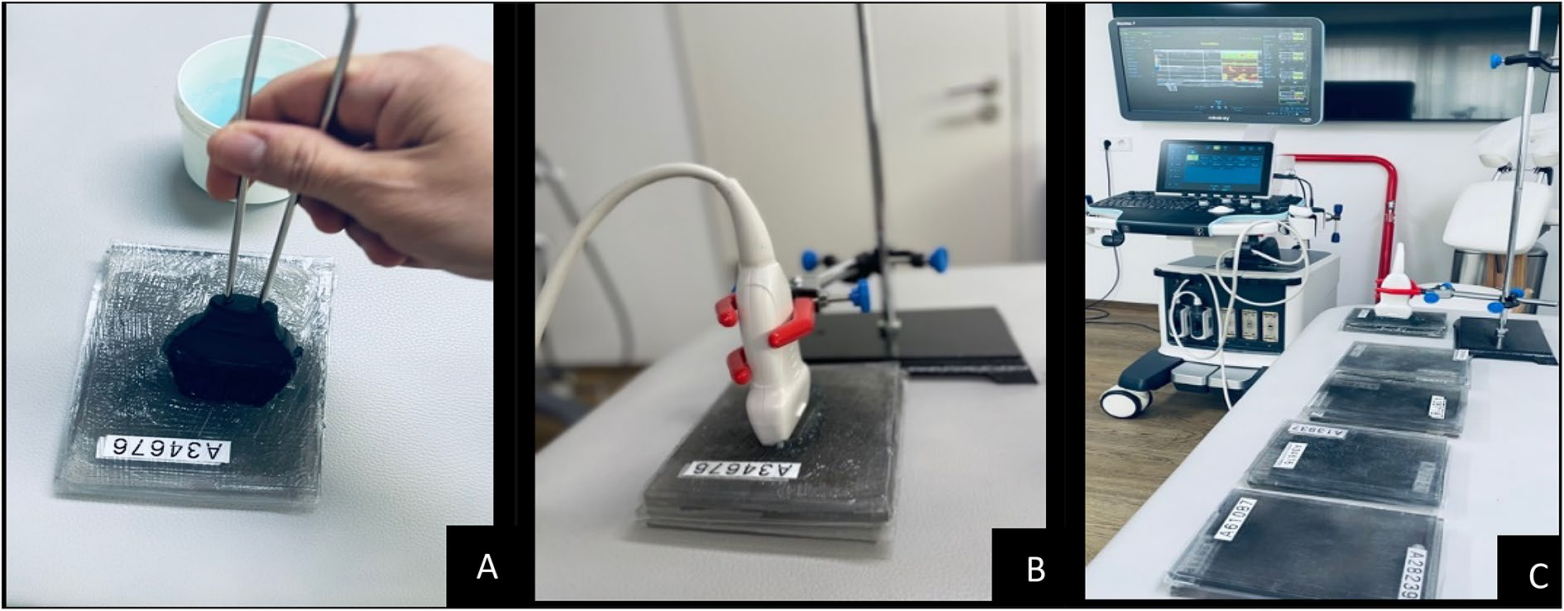
(**A**–**C**) Surface view of the SWE setup: (**A**) immersion in transmission gel with no applied pressure; (**B**) probe placement parallel to the phantom layers; and (**C**) stabilization with metallic holders to prevent motion and compression artifacts.

SWE measurements were performed using a high-resolution ultrasound device (Resona 7, Mindray Bio-Medical Electronics, China) equipped with a linear transducer (L11-3U, 3–11 MHz, 43 mm width). The system was operated under the musculoskeletal preset, with push pulse settings (Qgen) defined between 5.6 and 10 MHz. To ensure consistency and prevent compression artifacts, the transducer was mounted on a metallic holder in a parallel position relative to the phantom surface. A generous amount of transmission gel (Aquasonic 100, Parker Laboratories, USA) was applied to eliminate any probe pressure (Fig. 2A–C). All measurements were conducted in a no-contact configuration, which was selected to improve reliability by eliminating operator-induced variability and preventing deformation of the superficial gel layers, factors known to compromise the reproducibility of SWE data in contact-based protocols.

The imaging depth was standardized at 25 mm, and the elastography color scale was set to E3 with a stiffness range limited to 0–800 kPa. The Motion Stability Index (M-STB) feature was activated to optimize acquisition quality. For each tissue-mimicking layer, three separate acquisitions were obtained, focusing on the central zone of the sample to avoid edge artifacts. The region of interest (ROI) was manually adjusted to fully cover the layer’s anatomical width without including boundary transitions.

For each acquisition, the system provided SWV and corresponding E. These values were recorded as mean, minimum, maximum, and standard deviation.

#### MyotonPRO measurements

**Fig. 3:**
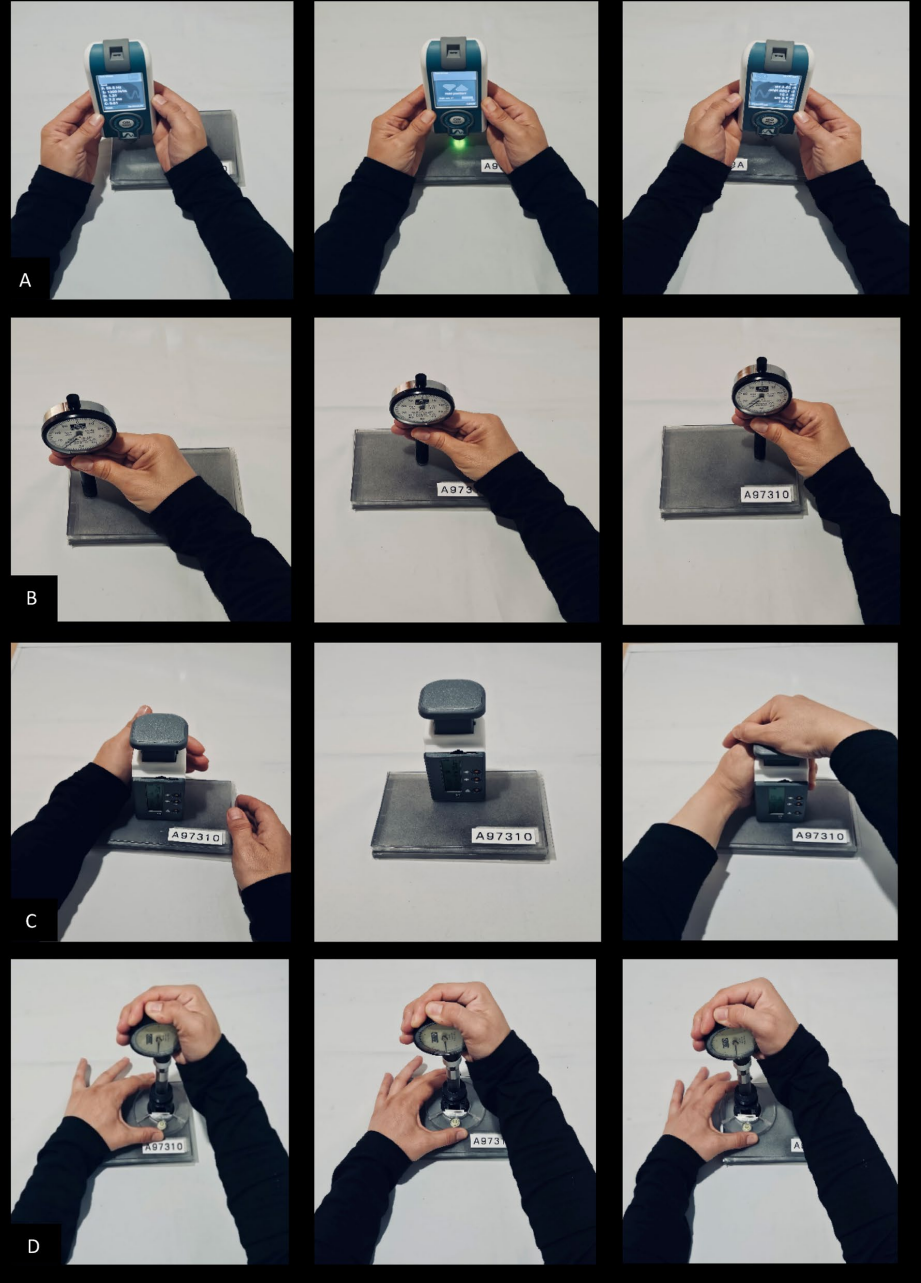
(**A**–**D**) Stiffness and compliance assessments of phantom tissues using four different devices. (**A**) MyotonPRO: triplicate measurements at middle, right, and left points. (**B**) Shore Durometer: triplicate readings from the same regions of each test site. (C) IndentoPRO: controlled-force stiffness assessment (10 N/s) with 10 mm indentations at three locations. (D) Tissue Compliance Meter (TCM): triplicate compliance measurements at the middle, right, and left regions.

The MyotonPRO (Myoton AS, Estonia) assesses dynamic stiffness (N/m) by delivering brief mechanical impulses through a 3 mm probe. Prior to each measurement, the probe was positioned perpendicularly to the phantom surface, and a stable preload of 0.18 N was ensured. This preload was standardized across operators using the device’s integrated visual cue: when the red light turned green, it indicated that the target preload had been consistently applied. The device then delivered a 0.4 N mechanical tap to induce local oscillations, which were captured by an internal accelerometer. Dynamic stiffness was automatically computed by the software based on the resulting oscillation waveforms^27^. Measurements were taken from three standardized locations (central, medial, and lateral) on each phantom layer (Fig. 3A). Data with a coefficient of variation (CV) > 3% were excluded to ensure measurement reliability, and the remaining valid values were averaged for analysis^33^.

#### Shore durometer measurements

Hardness measurements were obtained using the Shore Durometer (Type 1600-OO, Rex Gauge Co., Canada), with a 2 mm indenter tip. Three transverse locations (center, left, right) were tested per layer (Fig. 3B), with each indentation held for 1–2 s^53^. The device was applied vertically, under consistent manual pressure, and each indentation was allowed to stabilize for 1–2 s before recording the value. The device displays a stiffness value on an analog dial ranging from 0 to 100 (UI), where lower Shore-OOO values indicate softer materials^54^. Readings were averaged and recorded.

#### IndentoPRO measurements

The IndentoPRO device (Fascia Research Group, Ulm University; University of Chemnitz, Germany) features an 11.3 mm diameter compression probe, a Compression Load Cell FX1901 (TE Connectivity, Switzerland), and a ThinPot 10 kΩ membrane potentiometer (Spectra Symbol, USA)^44^. Force was applied manually at a controlled rate of 10 N/s until a 10 mm indentation was reached, indicated by an audible signal. Stiffness was calculated as the slope of the force–displacement curve and expressed in N/mm, with lower values reflecting softer tissue. Measurements were taken at the central, medial, and lateral regions of each phantom site. For each location, three consecutive trials were performed, and the average was used for analysis. Only trials with a coefficient of variation (CV) below 5% were included. Each phantom layer was tested at three locations (center, medial, lateral; Fig. 3C). Stiffness was calculated as the slope (N/mm) of the linear portion (2–8 mm) of the force–displacement curve. Trials with a coefficient of variation (CV) > 5% were excluded. The device was recalibrated every 45 min, and probe alignment was visually checked before each trial. Trained assessors, blinded to anatomical region and condition, completed > 30 pilot tests and followed a randomized measurement order^35^.

#### Tissue compliance meter (TCM) measurements

The Tissue Compliance Meter (TCM)^55^, is a validated instrument for assessing tissue compliance through standardized indentation. The system includes a 1 cm^2^ central probe within an 8 cm ring to ensure vertical alignment and reduce lateral force, which is crucial in fat-rich or uneven plantar areas. A gradual vertical load was applied until a 10 mm indentation depth was reached, while the required force (N) was continuously recorded using a built-in gauge (accuracy ± 0.05 N). This protocol is consistent with previous studies evaluating the plantar fat pad^46^. Each phantom layer was tested at three points (central, medial, lateral), and the mean was calculated. Trials with a CV > 5% were excluded. A 60-s rest was given between trials. All assessors performed over 30 pilot trials and were blinded to region and model. Measurement order was randomized. TCM’s large contact area and depth capacity make it suitable for assessing soft, fat-rich plantar tissues^37,46^.

### Blinding and randomization

Each phantom layer and assembled model was assigned a unique alphanumeric code using R software^56^, which masked information regarding the anatomical region, health condition (healthy or diabetic), and stiffness level. All coded samples were sealed in opaque, unlabeled packaging to conceal their internal configuration. This coding system was managed by a researcher independent from the assessment and analysis phases.

To ensure rigorous blinding, a double-blind protocol was implemented: assessors were blinded to the phantom type, region, and mechanical characteristics, while device operators were blinded to measurements obtained by other tools. Measurements for each device (SWE, MyotonPRO, Shore Durometer, IndentoPRO, and Tissue Compliance Meter) were independently randomized using a block randomization scheme, and spatial test points (central, medial, and lateral) were randomly varied across trials to account for intra-layer heterogeneity.

All measurements were conducted by two assessors with doctoral-level training in biomechanics and clinical physiotherapy, each with a minimum of 5 years of experience using quantitative stiffness assessment tools in both research and clinical settings. Prior to data collection, the assessors received standardized training on all device-specific protocols to ensure consistency. While basic operation of the measurement tools is accessible to trained physiotherapists, reproduction of the observed precision may require advanced methodological expertise and adherence to rigorous calibration and blinding protocols as described in this study.

### Statistical analysis

All statistical analyses were conducted using SPSS (v28.0; IBM) and R (v4.3.1; R Core Team, 2023), with significance set at *p* < 0.05. A blinded biostatistician performed all procedures independently. Data were screened for outliers (via z-scores and boxplots), missing values, and entry errors. No outliers were excluded, as all values fell within ± 3 SD. Normality was confirmed using Shapiro–Wilk tests, histograms, and Q–Q plots, allowing for parametric analyses. Intra- and inter-rater reliability were assessed using two-way mixed-effects intraclass correlation coefficients (ICC, absolute agreement), and agreement was visualized with Bland–Altman plots^57^. Concurent validity was evaluated using Pearson correlations between device-based stiffness and SWE-derived *E*. Predictive validity was assessed using linear regression analyses, reporting unstandardized beta coefficients (β), coefficient of determination (R^2^), standard errors (SE), and *p*-values. All regression assumptions (normality, homoscedasticity, independence of residuals) were satisfied; multicollinearity was excluded (variance inflation factor, VIF < 2). Between-group comparisons (healthy vs. diabetic) used independent-samples t-tests by region. One-way ANOVA with Bonferroni post hoc tests examined differences across stiffness configurations. Effect sizes were reported using Cohen’s d for pairwise comparisons^58^ and eta squared (η^2^) for ANOVA results. According to established guidelines, η^2^ values of 0.01, 0.06, and 0.14 correspond to small, medium, and large effects, respectively^59^. A multiple regression model also examined the combined effects of region, device, and configuration on stiffness.

## Results

### Inter- and ıntra-rater reproducibility

Table 3 presents the reproducibility results for the four stiffness measurement devices (MyotonPRO, Durometer, IndentoPRO, and TCM) based on inter- and intra-rater assessments. No statistically significant differences were observed between raters for any device (*p* > 0.05). Inter-rater reliability was excellent, with intraclass correlation coefficients (ICC) ranging from 0.979 (Durometer) to 0.994 (IndentoPRO). Similarly, intra-rater analyses revealed no significant test–retest differences across devices (*p* > 0.05), with consistently high ICC values: 0.966 (MyotonPRO), 0.969 (Durometer), 0.993 (IndentoPRO), and 0.992 (TCM), indicating strong reproducibility. The standard error of measurement (SEM) and minimal detectable change at 90% confidence (MDC90) are also reported in Table 3, further supporting the precision and stability of these tools.

**Table 3:** Assessment of intra- and inter-rater reproducibility for MyotonPRO, durometer, IndentoPRO, and TCM.

	Inter-rater reliability					
	Rater 1	Rater 2	*t* (*p*)	*ICC* (%95 CI)	SEM	MDC_90_
	*X* ± *SD*	*X* ± *SD*				
MyotonPRO stiffness (N/m)	1008.51 ± 57.63	1009.43 ± 57.61	− 0.875 (0.383)	0.986 (0.981; 0.990)*	1.59	3.49
Durometer shore-OOO (UI)	44.06 ± 4.75	44.15 ± 4.65	− 0.867 (0.387)	0.979 (0.971; 0.984)*	0.20	1.24
IndentoPRO depth-10 mm (N)	3.12 ± 1.05	3.15 ± 1.06	− 1.947 (0.052)	0.994 (0.992; 0.996)*	0.01	0.30
TCM-Depth-10 mm (N/mm)	32.67 ± 9.78	32.85 ± 9.39	− 1.236 (0.218)	0.990 (0.986; 0.992)*	0.20	1.23

	Intra-rater reliability					
	Test	Re-test	Test (p)	*ICC* (%95 GA)	SEM	MDC_90_
	*X* ± *SD*	*X* ± *SD*				
MyotonPRO stiffness (N/m)	1008.51 ± 57.63	1007.90 ± 57.25	0.376 (0.708)	0.966 (0.954; 0.975)*	3.79	5.40
Durometer shore-OOO (UI)	44.06 ± 4.75	44.21 ± 4.71	− 1.143 (0.255)	0.969 (0.957; 0.977)*	0.29	1.50
IndentoPRO depth-10 mm (N)	3.12 ± 1.05	3.12 ± 1.08	0.281 (0.779)	0.993 (0.990; 0.995)*	0.02	0.35
TCM-Depth-10 mm (N/mm)	32.67 ± 9.78	32.59 ± 9.43	0.607 (0.545)	0.992 (0.989; 0.994)*	0.15	1.07

*p < 0.05; Paired t Test (t); Intraclass Correlation Coefficient (ICC); Confidence Interval (CI); Standard Error of Measurement (SEM); Minimal Detectable Change (MDC); Descriptive statistics are presented as mean (${\overline{\text{X}}}$) and standard deviation (SD). Shore-OOO = Shore hardness scale in the OOO range; E (kPa) = Young’s modulus, expressed in kilopascals; SWV (m/s) = Shear wave velocity, in meters per second; ms = Milliseconds; N = Newton; N/m = Newton per meter; N/mm = Newton per millimeter; UI = Unit Indicator displayed on the device interface.

**Fig. 4:**
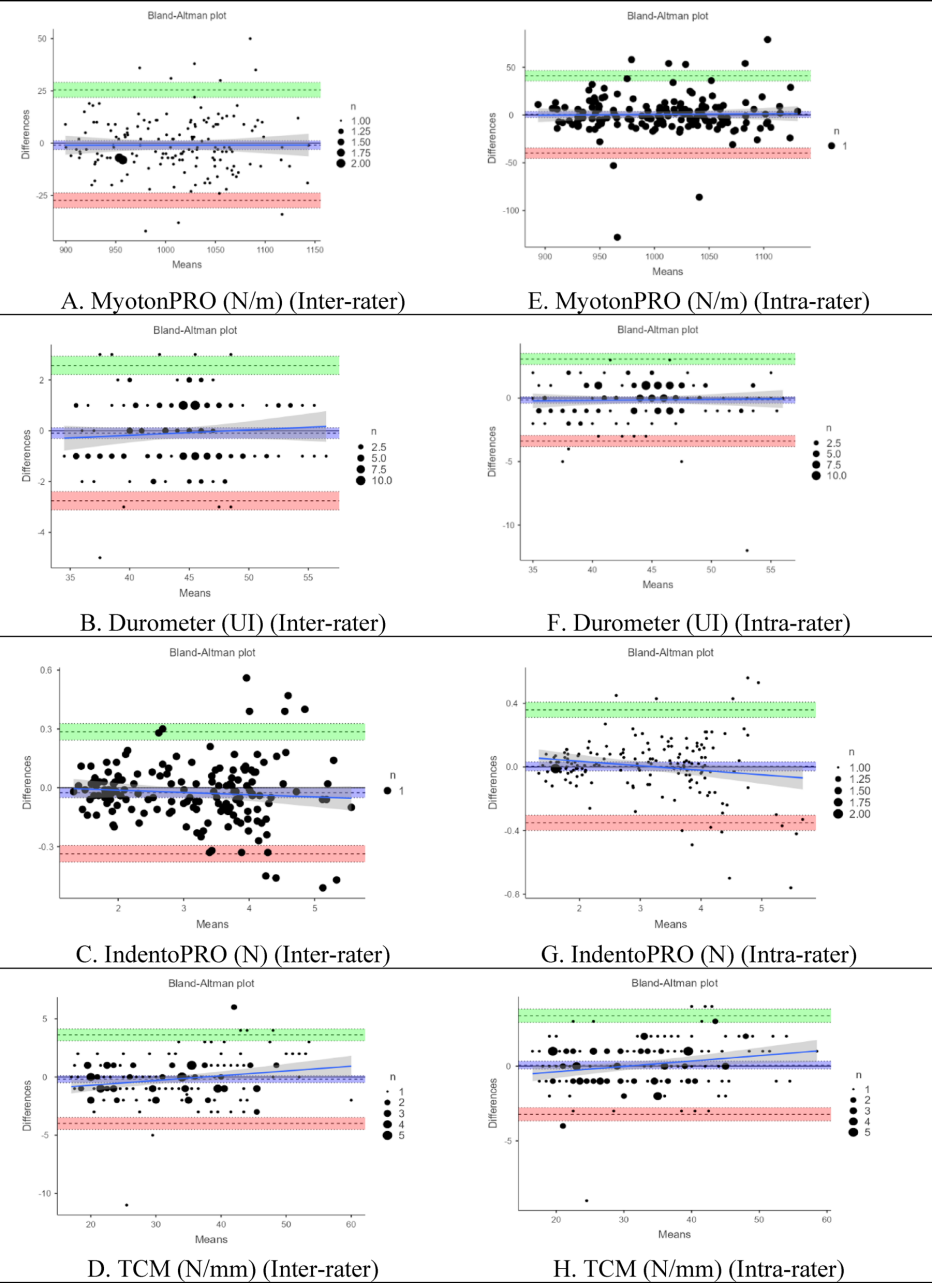
(**A**–**H**) Agreement Analysis of Stiffness Measurement Tools Using Bland–Altman Plots: Inter-Rater and Intra-Rater Comparisons. *Panels (**A**–**D**) show inter-rater, and Panels (**E**–**H**) show intra-rater agreement for MyotonPRO, Durometer, IndentoPRO, and TCM. Each plot displays the mean bias and 95% limits of agreement. Narrow limits and low bias reflect strong measurement consistency within and between raters.

Bland–Altman plots (Fig. 4A–H) were used to assess agreement across raters and repeated measurements. Panels A–D illustrate inter-rater agreement, and Panels E–H display intra-rater agreement for each device. All plots demonstrated narrow limits of agreement and minimal bias, indicating high measurement consistency.

### Regional comparison of stiffness in healthy and diabetic phantom models

**Fig. 5:**
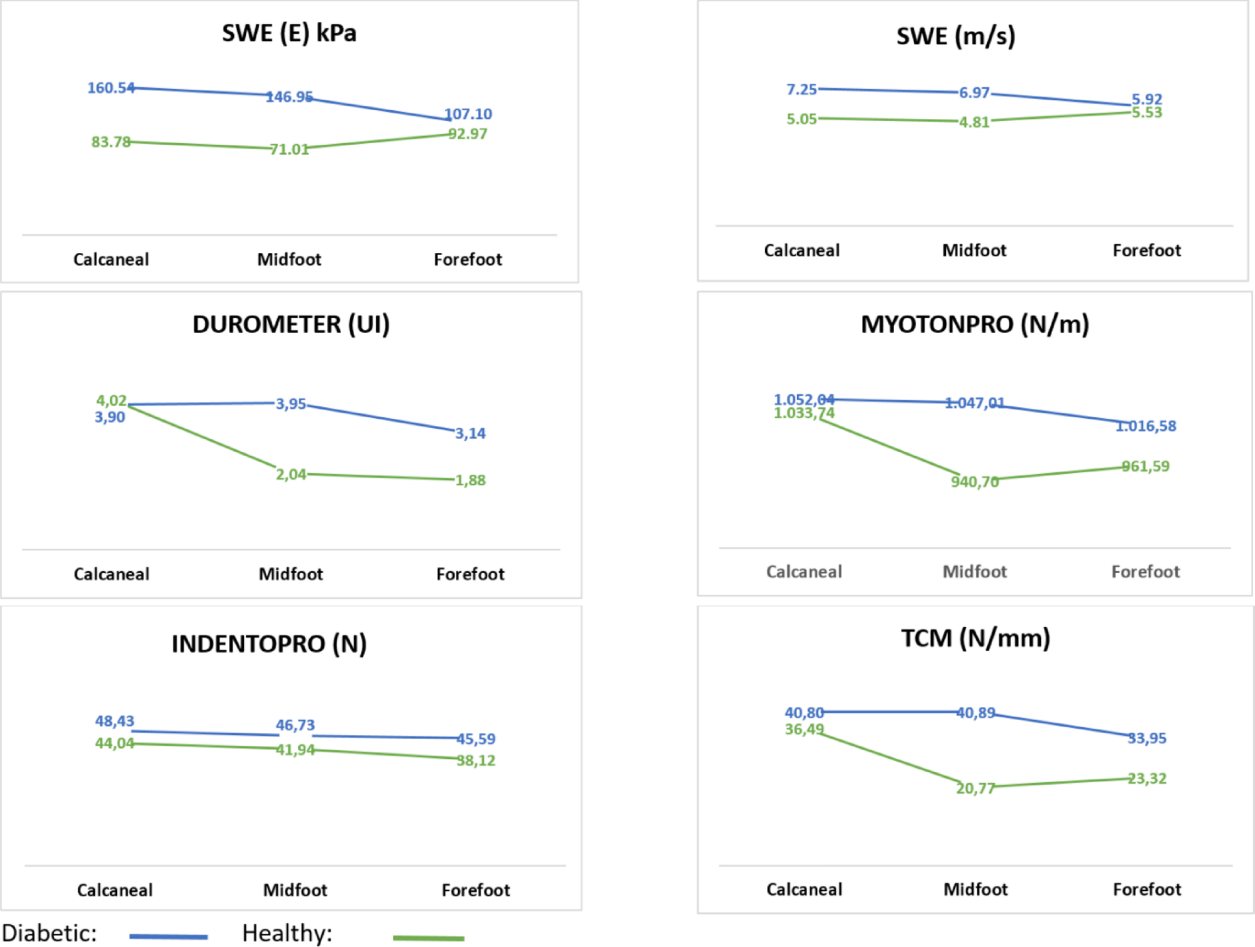
Regional comparison of SWE and mechanical stiffness measurements in healthy and diabetic plantar models. Line graphs illustrating regional differences in shear wave elastography (SWE) values—Young’s modulus (E, kPa) and shear wave velocity (m/s)—as well as mechanical stiffness measurements obtained using the Durometer (Shore-OO), MyotonPRO (N/m), IndentoPRO (N), and TCM (N/mm) across the calcaneal, midfoot, and forefoot regions. Data are presented separately for diabetic (blue line) and healthy (green line) phantom models.

Stiffness measurements obtained using SWE, MyotonPRO, Durometer, IndentoPRO, and TCM across the calcaneal, midfoot, and forefoot regions in healthy and diabetic phantom models are summarized in Table 4 and illustrated in Fig. 5.

**Table 4:** Comparison of SWE, MyotonPRO, durometer, IndentoPRO, and TCM measurements across plantar foot regions in healthy and diabetic phantom models.

Variable	Foot region	Group		Test statistics^†^	Group × foot region interaction
		Healthy	Diabetic		
		X ± SD	X ± SD		
SWE SWV (m/s)	Calcaneal^a^	5.05 ± 1.60	7.25 ± 0.99	*p* < 0.001* η^2^ = 0.31	F = 15.46 * p* < 0.001* η^2^ = 0.165
	Midfoot^b^	4.81 ± 0.73	6.97 ± 0.68	*p* < 0.001* η^2^ = 0.30	
	Forefoot^c^	5.53 ± 0.64	5.92 ± 0.81	*p* = 0.139 η^2^ = 0.01	
Test statistics^ϕ^		*p* = 0.022 η^2^ = 0.05	*p* < 0.001* η^2^ = 0.15		
		(a = b) > c	(a = b) > c		
SWE Young modulus (kPa)	Calcaneal^a^	83.78 ± 51.71	160.54 ± 43.99	*p* < 0.001* η^2^ = 0.29	F = 14.11 * p* < 0.001* η^2^ = 0.15
	Midfoot^b^	71.01 ± 26.47	146.95 ± 28.16	*p* < 0.001* η^2^ = 0.29	
	Forefoot^c^	92.97 ± 21.34	107.10 ± 29.16	*p* = 0.141 η^2^ = 0.01	
Test statistics^ϕ^		*p* = 0.073 η^2^ = 0.03	*p* < 0.001* η^2^ = 0.17		
Post Hoc		–	(a = b) > c		
MyotonPRO stiffness (N/m)	Calcaneal^a^	1033.74 ± 47.87	1052.04 ± 46.96	*p* = 0.075 η^2^ = 0.02	F = 18.78 * p* < 0.001* η^2^ = 0.19
	Midfoot^b^	940.70 ± 26.23	1047.01 ± 23.24	*p* < 0.001* η^2^ = 0.41	
	Forefoot^c^	961.59 ± 24.57	1016.58 ± 45.85	*p* < 0.001* η^2^ = 0.16	
Test statistics^ϕ^		*p* < 0.001* η^2^ = 0.37	*p* = 0.001* η^2^ = 0.08		
Post Hoc		a > (b = c)	(a = b) > c		
Durometer shore-OO (UI)	Calcaneal^a^	4.02 ± 0.88	3.90 ± 0.60	*p* = 0.440 η^2^ = 0.01	F = 43.55 * p* < 0.001* η^2^ = 0.36
	Midfoot^b^	2.04 ± 0.75	3.95 ± 0.22	*p* < 0.001* η^2^ = 0.49	
	Forefoot^c^	1.88 ± 0.23	3.14 ± 0.44	*p* < 0.001* η^2^ = 0.29	
Test statistics^ϕ^		*p* < 0.001* η^2^ = 0.60	*p* < 0.001* η^2^ = 0.18		
Post Hoc		a > (b = c)	(a = b) > c		
IndentoPRO depth-10 mm (N)	Calcaneal^a^	44.04 ± 4.85	48.43 ± 4.54	*p* < 0.001* η^2^ = 0.14	F = 3.71 * p* = 0.027* η^2^ = 0.05
	Midfoot^b^	41.94 ± 2.63	46.73 ± 1.90	*p* < 0.001* η^2^ = 0.16	
	Forefoot^c^	38.12 ± 2.02	45.59 ± 1.52	*p* < 0.001* η^2^ = 0.32	
Test statistics^ϕ^		*p* < 0.001* η^2^ = 0.23	*p* = 0.005* η^2^ = 0.07		
Post Hoc		a > (b = c)	(a = b) > c		
TCM-depth-10 mm (N/mm)	Calcaneal^a^	36.49 ± 8.53	40.80 ± 7.36	*p* = 0.003* η^2^ = 0.06	F = 31.47 * p* < 0.001* η^2^ = 0.29
	Midfoot^b^	20.77 ± 2.57	40.89 ± 3.20	*p* < 0.001* η^2^ = 0.56	
	Forefoot^c^	23.32 ± 2.20	33.95 ± 3.79	*p* < 0.001* η^2^ = 0.27	
Test statistics^ϕ^		*p* < 0.001* η^2^ = 0.48	*p* < 0.001* η^2^ = 0.17		
Post Hoc		a > (b = c)	(a = b) > c		

*p < 0.05; Mixed ANOVA (F); Effect Size (η^2^); Bonferroni–Dunn Test (a. b. c); ϕ: within-group comparisons; †: between-group comparisons; Descriptive statistics are presented as mean (X̄) and standard deviation (SD). Shore-OOO = Shore hardness scale in the OOO range; E (kPa) = Young’s modulus, expressed in kilopascals; SWV (m/s) = Shear wave velocity, in meters per second; msec = Milliseconds; N = Newton; N/m = Newton per meter; N/mm = Newton per millimeter; UI = Unit Indicator displayed on the device interface.

For SWE, shear wave velocity (SWV) values were significantly higher in diabetic models in the calcaneal (*p* < 0.001, η^2^ = 0.31) and midfoot (*p* < 0.001, η^2^ = 0.30) regions, with no significant difference in the forefoot (*p* = 0.139). Similarly, Young’s modulus (E) values were significantly elevated in diabetic models in the calcaneal and midfoot regions (both *p* < 0.001), but not in the forefoot (*p* = 0.141). Mixed ANOVA revealed significant main and interaction effects for both SWV and Young’s modulus (*p* < 0.001), with post hoc tests confirming greater stiffness in the calcaneal and midfoot regions compared to the forefoot.

For MyotonPRO, significant group differences were observed in the midfoot (*p* < 0.001, η^2^ = 0.41) and forefoot (*p* < 0.001, η^2^ = 0.16), but not in the calcaneal region (*p* = 0.075). Both within-group and interaction effects were significant (*p* < 0.001). Post hoc analysis showed that in healthy models, stiffness followed the pattern calcaneal > midfoot = forefoot, while in diabetic models, the pattern was calcaneal = midfoot > forefoot.

Durometer measurements showed significantly higher stiffness in diabetic models for the midfoot and forefoot regions (*p* < 0.001), but not for the calcaneal region (*p* = 0.440). Main and interaction effects were significant (*p* < 0.001). The regional pattern in healthy models was calcaneal > midfoot = forefoot, whereas in diabetic models it was calcaneal = midfoot > forefoot.

IndentoPRO-based force values (at 10 mm indentation depth) were significantly greater in diabetic models across all three regions (*p* < 0.001). Both regional differences and interaction effects were statistically significant (*p* < 0.001 and *p* = 0.027, respectively). The stiffness distribution was calcaneal > midfoot = forefoot in healthy models and calcaneal = midfoot > forefoot in diabetic models.

For TCM, all stiffness values were significantly elevated in diabetic models across regions (calcaneal: *p* = 0.003; midfoot and forefoot: *p* < 0.001). A strong grou* p* × region interaction was observed (*p* < 0.001), and regional patterns were consistent with other tools: calcaneal > midfoot = forefoot in healthy models, and calcaneal = midfoot > forefoot in diabetic models.

### Regression analyses with SWE parameters

Table 5 presents the regression results evaluating the predictive value of each device’s stiffness measurements for SWV. All four devices showed statistically significant associations with SWV in the calcaneal region for both healthy and diabetic models (*p* < 0.001). Notably, TCM demonstrated the strongest association in the diabetic forefoot region (β = 0.155, R^2^ = 0.527). In the pooled analysis across all regions and models, TCM measurements explained nearly half of the variance in SWV (β = 0.097, R^2^ = 0.483), followed by IndentoPRO (R^2^ = 0.315), MyotonPRO (R^2^ = 0.307), and Durometer (R^2^ = 0.285).

**Table 5:** Regression analysis of stiffness measurement tools on shear wave velocity in plantar soft tissue.

Variable	Foot region	Group	Velocity (m/s)		Statistical significance of the model		
			*β* (%95 CI)	*r*	*F*	*p*	*R*^2^
MyotonPRO stiffness (N/m)	Calcaneal	Healthy	0.021 (0.010; 0.032)	0.628	16.286	< 0.001**	0.394
		Diabetic	0.013 (0.007; 0.020)	0.640	17.317	< 0.001**	0.409
	Midfoot	Healthy	0.010 (− 0.001; 0.021)	0.375	4.080	0.054	0.140
		Diabetic	0.009 (− 0.003; 0.020)	0.304	2.552	0.123	0.093
	Forefoot	Healthy	0.012 (0.002; 0.021)	0.446	6.212	0.020*	0.199
		Diabetic	− 0.008 (− 0.015; − 0.002)	− 0.461	6.749	0.016*	0.213
	All measurements	0.013 (0.010; 0.016)	0.554	70.915	< 0.001**	0.307	
Durometer shore-OO (UI)	Calcaneal	Healthy	1.191 (0.629; 1.752)	0.658	19.094	< 0.001**	0.433
		Diabetic	1.276 (0.853; 1.699)	0.779	38.596	< 0.001**	0.607
	Midfoot	Healthy	0.056 (− 0.345; 0.456)	0.057	0.082	0.777	0.003
		Diabetic	0.773 (− 0.459; 2.005)	0.250	1.668	0.208	0.063
	Forefoot	Healthy	1.243 (0.219; 2.267)	0.447	6.248	0.019*	0.200
		Diabetic	1.079 (0.468; 1.689)	0.588	13.245	0.001*	0.346
	All measurements	0.664 (0.500; 0.828)	0.534	63.902	< 0.001**	0.285	
IndentoPRO depth-10 mm (N)	Calcaneal	Healthy	0.157 (0.037; 0.277)	0.476	7.309	0.012*	0.226
		Diabetic	0.101 (0.022; 0.180)	0.466	6.932	0.014*	0.217
	Midfoot	Healthy	0.135 (0.036; 0.234)	0.489	7.844	0.010*	0.239
		Diabetic	0.089 (− 0.054; 0.232)	0.249	1.649	0.211	0.062
	Forefoot	Healthy	0.14 (0.023; 0.257)	0.441	6.036	0.021*	0.194
		Diabetic	− 0.102 (− 0.318; 0.113)	− 0.192	0.960	0.337	0.037
	All measurements	0.16 (0.123; 0.197)	0.561	73.498	< 0.001**	0.315	
TCM-depth-10 mm (N/mm)	Calcaneal	Healthy	0.134 (0.081; 0.188)	0.717	26.417	< 0.001**	0.514
		Diabetic	0.104 (0.07; 0.139)	0.779	38.517	< 0.001**	0.606
	Midfoot	Healthy	0.083 (− 0.028; 0.194)	0.295	2.374	0.136	0.087
		Diabetic	0.061 (− 0.023; 0.145)	0.287	2.247	0.146	0.082
	Forefoot	Healthy	0.179 (0.084; 0.274)	0.615	15.191	0.001*	0.378
		Diabetic	0.155 (0.094; 0.215)	0.726	27.832	< 0.001**	0.527
	All measurements	0.097 (0.081; 0.112)	0.695	149.682	< 0.001**	0.483	

*p < 0.05; **p < 0.01; Regression Analysis (F); Regression Coefficient (β); Pearson Correlation Coefficient (r); Coefficient of Determination (R^2^); Confidence Interval (CI). Shore-OOO = Shore hardness scale in the OOO range; E (kPa) = Young’s modulus, expressed in kilopascals; SWV (m/s) = Shear wave velocity, in meters per second; msec = Milliseconds; N = Newton; N/m = Newton per meter; N/mm = Newton per millimeter; UI = Unit Indicator displayed on the device interface.

Table 6 summarizes the regression outcomes for mechanical stiffness values in predicting SWE-derived *E.* All four devices demonstrated significant associations with *E* in the calcaneal region of both healthy and diabetic models (*p* ≤ 0.001). In the forefoot region, TCM again exhibited the strongest predictive relationship, particularly in the diabetic model (β = 5.55, R^2^ = 0.520). No statistically significant associations were observed in the midfoot region for any device. In the full regional model, TCM demonstrated the highest explanatory power (R^2^ = 0.508), followed by Durometer (R^2^ = 0.317), IndentoPRO (R^2^ = 0.315), and MyotonPRO (R^2^ = 0.315).

**Table 6:** Regression analysis of stiffness measurement tools on SWE-derived elastic modulus in plantar soft tissue.

Variable	Foot region	Group	*β* (%95 CI)	*r*	Statistical significance of the model		
					*F*	*p*	*R*^*2*^
MyotonPRO stiffness (N/m)	Calcaneal	Healthy	0.630 (0.269; 0.992)	0.583	12.902	0.001*	0.340
		Diabetic	0.581 (0.278; 0.883)	0.620	15.609	0.001*	0.384
	Midfoot	Healthy	0.321 (− 0.073; 0.715)	0.318	2.819	0.106	0.101
		Diabetic	0.376 (− 0.099; 0.851)	0.310	2.661	0.115	0.096
	Forefoot	Healthy	0.389 (0.069; 0.709)	0.448	6.268	0.019*	0.200
		Diabetic	− 0.31 (− 0.539; − 0.081)	− 0.487	7.781	0.010*	0.237
	All measurements		0.474 (0.365; 0.584)	0.561	73.673	< 0.001**	0.315
Durometer shore-OO (UI)	Calcaneal	Healthy	38.358 (20.186; 56.531)	0.656	18.899	< 0.001**	0.431
		Diabetic	56.508 (37.431; 75.585)	0.773	37.217	< 0.001**	0.598
	Midfoot	Healthy	1.727 (− 12.886; 16.339)	0.049	0.059	0.810	0.002
		Diabetic	34.93 (− 15.614; 85.473)	0.274	2.026	0.167	0.075
	Forefoot	Healthy	41.322 (7.217; 75.427)	0.447	6.227	0.020*	0.199
		Diabetic	38.86 (16.809; 60.91)	0.587	13.173	0.001*	0.345
	All measurements		25.257 (19.467; 31.048)	0.563	74.199	< 0.001**	0.317
IndentoPRO depth-10 mm (N)	Calcaneal	Healthy	4.356 (0.348; 8.365)	0.409	5.010	0.034*	0.167
		Diabetic	4.352 (0.79; 7.915)	0.450	6.331	0.019*	0.202
	Midfoot	Healthy	4.356 (0.627; 8.085)	0.434	5.787	0.024*	0.188
		Diabetic	3.808 (− 2.08; 9.697)	0.257	1.774	0.195	0.066
	Forefoot	Healthy	4.642 (0.741; 8.542)	0.440	6.008	0.022*	0.194
		Diabetic	− 3.939 (− 11.688; 3.81)	− 0.205	1.096	0.305	0.042
	All measurements		5.793 (4.458; 7.127)	0.561	73.467	< 0.001**	0.315
TCM-depth-10 mm (N/mm)	Calcaneal	Healthy	4.232 (2.445; 6.019)	0.698	23.780	< 0.001**	0.487
		Diabetic	4.686 (3.158; 6.214)	0.784	39.904	< .001**	0.615
	Midfoot	Healthy	2.41 (− 1.714; 6.534)	0.234	1.449	0.240	0.055
		Diabetic	2.672 (− 0.782; 6.127)	0.304	2.539	0.124	0.092
	Forefoot	Healthy	6.015 (2.882; 9.149)	0.620	15.632	0.001*	0.385
		Diabetic	5.554 (3.357; 7.751)	0.721	27.097	< 0.001**	0.520
	All measurements		3.586 (3.035; 4.136)	0.713	165.319	< 0.001**	0.508

*p < 0.05; **p < 0.01; Regression Analysis (F); Regression Coefficient (β); Pearson Correlation Coefficient (r); Coefficient of Determination (R^2^); Confidence Interval (CI). Shore-OOO = Shore hardness scale in the OOO range; E (kPa) = Young’s modulus, expressed in kilopascals; SWV (m/s) = Shear wave velocity, in meters per second; msec = Milliseconds; N = Newton; N/m = Newton per meter; N/mm = Newton per millimeter; UI = Unit Indicator displayed on the device interface.

## Discussion

### Validity and reliability of mechanical devices using phantom models

This study demonstrated that multilayer polyurethane foot phantoms can effectively simulate the mechanical behavior of healthy and diabetic plantar soft tissues. Constructed using literature-based stiffness values and anatomical segmentation, these models enabled a standardized evaluation of mechanical measurement tools. Consistent with our hypothesis, stiffness measurements obtained from all four mechanical devices were significantly correlated with SWE-derived values, supporting their concurrent validity. Additionally, all devices exhibited high intra- and inter-rater reliability under blinded and standardized testing conditions (ICC ≥ 0.96), indicating strong repeatability under phantom-based conditions. However, their direct applicability to biological tissues remains uncertain due to the greater variability in vivo.

These findings align with previous reports on the reliability of MyotonPRO^33,36,43,60,61^, IndentoPRO^35,44,46^, and TCM^37,45,46^. Minimal variability across repeated measures (< 5%) further supports the usability of these tools in consistent clinical or research settings. IndentoPRO exhibited the highest raw correlation (r ≈ 0.9) with SWE-derived stiffness, consistent with Bartsch et al.^35^, indicating that force–displacement metrics from controlled indentation closely mirror the elastic modulus provided by elastography. TCM also showed strong associations (r ≈ 0.84) and emerged as the best predictor of SWE-based *E* values, potentially due to its deeper, fixed-depth indentation. In contrast, MyotonPRO and Shore Durometer, primarily assessing superficial tissue properties^31,36,46^, demonstrated slightly lower but still acceptable correlations (r ≈ 0.78–0.87). Taken together, these results suggest that each device has specific strengths depending on the depth and anatomical location of the target tissue. IndentoPRO and TCM appear particularly sensitive to deeper structures such as the heel pad, while MyotonPRO and the Shore Durometer provide portable and practical options for superficial tissues like the forefoot skin and subcutaneous layers. In addition to technical accuracy, factors such as portability, cost, and ease of use should also inform device selection in applied settings.

### Anatomical and regional variations in stiffness assessment

Our phantom models successfully reproduced region-specific stiffness patterns consistent with SWE findings in diabetic patients^29,62^. The heel and midfoot regions of diabetic phantoms exhibited higher stiffness than healthy models, simulating the known fibrotic remodeling and glycation-induced rigidity characteristic of diabetic foot pathology^63^. These regional stiffness increases are not the result of spontaneous biological change but are embedded by design, reflecting intentional differences in material modulus. Consequently, device-detected variations serve to verify measurement sensitivity and responsiveness rather than track disease progression. While these regional differences in stiffness are design-driven and not reflective of biological remodeling, they serve as a necessary foundation for evaluating the measurement accuracy and discriminatory capacity of the mechanical devices under standardized conditions. Although this outcome may appear expected, demonstrating that the devices can distinguish known, preconfigured stiffness gradients is essential for establishing their concurrent validity and reliability in detecting clinically relevant variations.

**Fig. 6:**
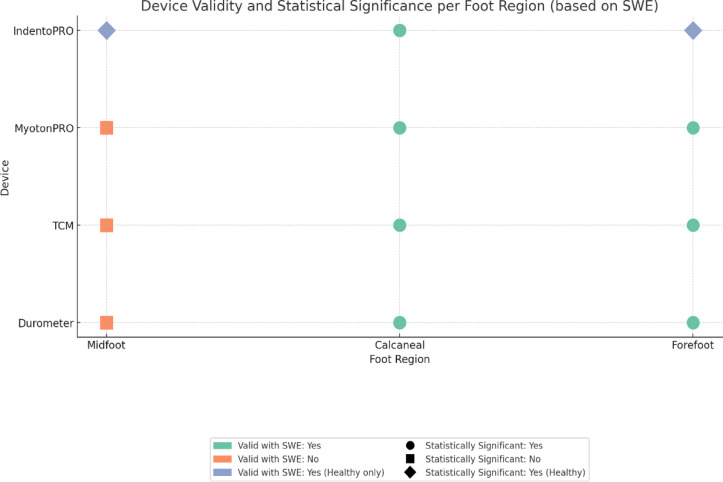
Region-specific measurement strengths of the four stiffness assessment tools across plantar foot. A comparative overview of the region-specific performance characteristics of MyotonPRO, Durometer, IndentoPRO, and TCM based on multilayer phantom model analysis. The figure summarizes optimal assessment zones, heel (calcaneal), midfoot, and forefoot, for each tool, considering their mechanical sensitivity and correlation with SWE. The visualization supports an integrated approach to diabetic foot stiffness assessment by guiding tool selection based on anatomical region and measurement capability.

Interestingly, the forefoot region remained relatively compliant in diabetic phantoms, reflecting clinical findings that suggest later or less pronounced degenerative changes in this area^64,65^. Studies on the plantar fascia support this pattern, noting proximal stiffening near the heel and preserved compliance in the forefoot^66–70^. This region-specific approach (Fig. 6) reinforces the idea that diabetic plantar stiffening is not a uniform process and that device selection should be guided by anatomical and biomechanical context.

The midfoot, in particular, emerged as a biomechanically complex region, where all devices exhibited weaker correlations with SWE and greater measurement variability. This finding is consistent with previous literature highlighting the anatomical intricacies and heterogeneous load-bearing structures of the midfoot, including intrinsic muscles and overlapping soft tissue layers^6,9,68,71^. Consistent with our findings, Tonna et al.^31^ also emphasized the difficulty of obtaining reliable stiffness measurements in this region. Our phantom models incorporated structural layering (skin, fat pad, fascia, muscle) but did not replicate internal anisotropy or directional fiber orientation, especially within the muscle layer. In addition, the flat phantom geometry limited the replication of the three-dimensional curvature and pressure distribution of the human foot, which may have contributed to estimation errors in the midfoot region. Future research should therefore focus on fabricating anatomically shaped phantom patches with tunable viscoelastic properties to enhance structural fidelity and improve translational relevance. Finite element modeling by Chatzistergos et al.^72^, supports the use of curved phantoms or imaging integration to improve measurement precision in this region.

### Clinical ımplications, limitations, and future directions

Region-specific mechanical stiffness assessments offer promising applications for diabetic foot screening and early risk detection. The phantom models used in this study successfully simulated diabetic foot stiffness patterns, particularly increased stiffness in the heel and midfoot, supporting their relevance for preclinical evaluation. Devices such as the TCM and IndentoPRO appear well-suited for deeper tissue assessment, while the MyotonPRO and Shore Durometer offer practical utility for evaluating more superficial layers.

Nevertheless, several limitations must be acknowledged. Although the phantom models were carefully constructed, they cannot replicate key biological features such as vascular perfusion, anisotropic fiber architecture, or dynamic viscoelasticity. Their flat geometry and uniform density assumptions may have influenced load distribution, particularly in the midfoot, while the fixed measurement depths of some devices may have introduced estimation errors. A further limitation of this study is related to the midfoot region. Despite the use of anatomically realistic phantom models, the correlations between some mechanical devices and SWE were lower in this area. This finding is consistent with previous research, where the structural complexity and heterogeneous composition of the midfoot have been associated with reduced measurement stability and reproducibility. Our results therefore indicate that, while phantom-based models improve standardization, they do not fully resolve the challenges of midfoot assessment, and future studies should explore optimized protocols or device adaptations for this region. Another limitation is that the stiffness values used to fabricate the phantom models were derived from previous literature, without direct in vivo comparisons. Although these references provided essential ranges for simulating healthy and diabetic conditions, the absence of concurrent in vivo validation may restrict the immediate clinical generalizability of our findings.

Future work should therefore integrate direct in vivo assessments to confirm and expand upon the phantom-based results. To improve translational applicability, studies should also focus on the development of anatomically shaped phantoms with tunable viscoelastic properties and on evaluating device responsiveness to clinical interventions. Integrating mechanical stiffness data with plantar pressure mapping and neuropathy screening could provide a more comprehensive risk profile for diabetic foot complications. Combining experimental phantom studies with computational models may further improve predictive accuracy and translational value. The integration of numerical simulations with experimental data could provide more comprehensive insights into plantar tissue mechanics and enhance the clinical applicability of stiffness measurement tools.

## Conclusion

This study demonstrated that four mechanical devices (MyotonPRO, Shore Durometer, IndentoPRO and TCM) are reliable and valid tools for assessing plantar soft tissue stiffness using multilayer polyurethane phantom models simulating healthy and diabetic foot conditions. All devices showed excellent intra- and inter-rater reliability and significant correlations with SWE-derived reference values, supporting their concurrent validity. Each device exhibited distinct strengths depending on tissue depth: the TCM and IndentoPRO were particularly effective in assessing deeper tissue stiffness, while the MyotonPRO and Shore Durometer were better suited for evaluating more superficial layers. Although phantom models cannot fully simulate the complexity of biological tissues, they offer a reproducible and standardized platform for evaluating device performance across anatomically relevant regions. This phantom-based approach may also support early-phase medical device evaluation, particularly in situations where in vivo testing is not immediately feasible. Moving forward, enhancing anatomical realism, validating findings in clinical settings, and integrating these tools with complementary diagnostics will be essential for advancing their clinical utility in diabetic foot care.

## Supplementary Information

Below is the link to the electronic supplementary material.

Supplementary Material 1

## Data Availability

The datasets generated and analyzed during this study are available from the corresponding author upon reasonable request.

## References

[1] International Diabetes Federation. *IDF Diabetes Atlas* 11th edn. (International Diabetes Federation, 2025).

[2] He, Q. et al. Global burden of type 2 diabetes in non-elderly individuals 1990 to 2021 and projections for 2050: A systematic analysis of the 2021 Global Burden of Disease. *Diabetes Metab.* **51**, 101660 (2025). 40348179 10.1016/j.diabet.2025.101660

[3] Gefen, A. Plantar soft tissue loading under the medial metatarsals in the standing diabetic foot. *Med. Eng. Phys.* **25**, 491–499 (2003). 12787987 10.1016/s1350-4533(03)00029-8

[4] Ledoux, W. R. et al. The association between mechanical and biochemical/histological characteristics in diabetic and non-diabetic plantar soft tissue. *J. Biomech.* **49**, 3328–3333 (2016). 27623704 10.1016/j.jbiomech.2016.08.021 PMC5074896

[5] Naemi, R. et al. Can plantar soft tissue mechanics enhance prognosis of diabetic foot ulcer?. *Diabetes Res. Clin. Pract.* **126**, 182–191 (2017). 28259007 10.1016/j.diabres.2017.02.002

[6] Wang, Y. N. et al. Histomorphological and biochemical properties of plantar soft tissue in diabetes. *Foot* **33**, 1–6 (2017). 29126035 10.1016/j.foot.2017.06.001 PMC5937986

[7] Ahmed, N. Advanced glycation endproducts—Role in pathology of diabetic complications. *Diabetes Res. Clin. Pract.* **67**, 3–21 (2005). 15620429 10.1016/j.diabres.2004.09.004

[8] Singh, V. P. et al. Advanced glycation end products and diabetic complications. *Korean J. Physiol. Pharmacol.* **18**, 1–14 (2014). 24634591 10.4196/kjpp.2014.18.1.1 PMC3951818

[9] Wang, Y. N., Lee, K. & Ledoux, W. R. Histomorphological evaluation of diabetic and non-diabetic plantar soft tissue. *Foot Ankle Int.* **32**, 802–810 (2011). 22049867 10.3113/FAI.2011.0802 PMC4227595

[10] Giacomozzi, C. et al. Does the thickening of Achilles tendon and plantar fascia contribute to the alteration of diabetic foot loading?. *Clin. Biomech.* **20**, 532–539 (2005). 10.1016/j.clinbiomech.2005.01.011 15836941

[11] Gelber, J. R. et al. Windlass mechanism in individuals with diabetes mellitus, peripheral neuropathy, and low medial longitudinal arch height. *Foot Ankle Int.* **35**, 816–824 (2014). 24917647 10.1177/1071100714538416 PMC4262736

[12] Klaesner, J. W. et al. Plantar tissue stiffness in patients with diabetes mellitus and peripheral neuropathy. *Arch. Phys. Med. Rehabil.* **83**, 1796–1801 (2002). 12474190 10.1053/apmr.2002.35661

[13] Sun, J. H. et al. Changes in the thickness and stiffness of plantar soft tissues in people with diabetic peripheral neuropathy. *Arch. Phys. Med. Rehabil.* **92**, 1484–1489 (2011). 21762874 10.1016/j.apmr.2011.03.015

[14] Ledoux, W. R. & Blevins, J. J. The compressive material properties of the plantar soft tissue. *J. Biomech.* **40**, 2975–2981 (2007). 17433335 10.1016/j.jbiomech.2007.02.009

[15] Yang, X. G. et al. A narrative review of the measurement methods for biomechanical properties of plantar soft tissue in patients with diabetic foot. *Front. Endocrinol.* **15**, 1332032 (2024). 10.3389/fendo.2024.1332032 PMC11317276 39135623

[16] Kumar, C. G. S. et al. Intrinsic foot muscle and plantar tissue changes in type 2 diabetes mellitus. *J. Diabetes* **7**, 850–857 (2015). 25496489 10.1111/1753-0407.12254

[17] Hsu, T. C. et al. Altered heel-pad mechanical properties in patients with type 2 diabetes mellitus. *Diabet. Med.* **17**, 854–859 (2000). 11168328 10.1046/j.1464-5491.2000.00394.x

[18] Dixit, R., Singh, S. & Garg, S. Evaluation of the plantar fascia in patients with diabetes mellitus: The role of sonoelastography. *Pol. J. Radiol.* **87**, 500–505 (2022). 10.5114/pjr.2022.119474 PMC9536209 36250143

[19] Saroha, A. et al. Ultrasonographic evaluation of thickness and stiffness of Achilles tendon and plantar fascia in type 2 diabetics patients: A cross-sectional observation study. *J. Med. Ultrasound* **31**, 282–286 (2023). 38264597 10.4103/jmu.jmu_109_22 PMC10802861

[20] Bus, S. A. et al. Intrinsic muscle atrophy and toe deformity in the diabetic neuropathic foot: A magnetic resonance imaging study. *Diabetes Care* **25**, 1444–1450 (2002). 12145248 10.2337/diacare.25.8.1444

[21] Haelewijn, N. et al. Test–retest and inter-rater reliability of intrinsic and extrinsic foot muscles using 2D ultrasound. In *Presented at the Int. Foot Ankle Biomechnical Society Congress* (2023).

[22] Morrison, T. et al. Can ultrasound measures of intrinsic foot muscles and plantar soft tissues predict future diabetes-related foot disease? A systematic review. *PLoS One* **13**, e0199055 (2018). 29906277 10.1371/journal.pone.0199055 PMC6003689

[23] Severinsen, K. et al. Atrophy of foot muscles in diabetic patients can be detected with ultrasonography. *Diabetes Care* **30**, 3053–3057 (2007). 17717286 10.2337/dc07-0108

[24] Wang, X. et al. Early detection of atrophy of foot muscles in Chinese patients of type 2 diabetes mellitus by high-frequency ultrasonography. *J. Diabetes Res.* **2014**, 927069 (2014). 25165725 10.1155/2014/927069 PMC4140103

[25] Andersen, H., Gjerstad, M. D. & Jakobsen, J. Atrophy of foot muscles: A measure of diabetic neuropathy. *Diabetes Care* **27**, 2382–2385 (2004). 15451904 10.2337/diacare.27.10.2382

[26] Thomas, V. J. et al. The role of skin hardness, thickness, and sensory loss on standing foot power in the development of plantar ulcers in patients with diabetes mellitus—A preliminary study. *Int. J. Low. Extrem. Wounds* **2**, 132–139 (2003). 15866837 10.1177/1534734603258601

[27] Banerjee, S. S. et al. A method to analyze plantar stiffness variation in diabetes using myotonometric measurements. *J. Med. Devices* **14**, 011105 (2020).

[28] Lin, C. Y. et al. Heel pad stiffness in plantar heel pain by shear wave elastography. *Ultrasound Med. Biol.* **41**, 2890–2898 (2015). 26299685 10.1016/j.ultrasmedbio.2015.07.004

[29] Naemi, R. et al. Diabetes status is associated with plantar soft tissue stiffness measured using ultrasound reverberant shear wave elastography approach. *J. Diabetes Sci. Technol.* **16**, 478–490 (2022). 33095039 10.1177/1932296820965259 PMC8861805

[30] Bezek, L. B. et al. Mechanical properties of tissue-mimicking composites formed by material jetting additive manufacturing. *J. Mech. Behav. Biomed. Mater.* **125**, 104938 (2022). 34740012 10.1016/j.jmbbm.2021.104938

[31] Madsen, E. L., Zagzebski, J. A. & Frank, G. R. Oil-in-gelatin dispersions for use as ultrasonically tissue-mimicking materials. *Ultrasound Med. Biol.* **8**, 277–287 (1982). 7101576 10.1016/0301-5629(82)90034-5

[32] Tejo-Otero, A. et al. Soft-tissue-mimicking using hydrogels for the development of phantoms. *Gels* **8**, 40 (2022). 35049575 10.3390/gels8010040 PMC8774477

[33] Liu, Y. & Maruvada, S. Development and characterization of polyurethane-based tissue and blood mimicking materials for high intensity therapeutic ultrasound. *J. Acoust. Soc. Am.* **151**, 3043–3051 (2022). 35649924 10.1121/10.0010385

[34] McGarry, C. K. et al. Tissue mimicking materials for imaging and therapy phantoms: A review. *Phys. Med. Biol.* **65**, 2301 (2020). 10.1088/1361-6560/abbd17 32998112

[35] Singh, S. et al. Recent advancements in polyurethane-based tissue engineering. *ACS Appl. Bio Mater.* **6**, 327–348 (2023). 36719800 10.1021/acsabm.2c00788

[36] Alfuraih, A. M. I. *Shear Wave Elastography in the Assessment of Healthy and Diseased Skeletal Muscle* (University of Leeds, 2019).

[37] Tonna, R. et al. Reliability and validity of shore hardness in plantar soft tissue biomechanics. *Sensors* **24**, 539 (2024). 38257632 10.3390/s24020539 PMC10818800

[38] Saldıran, T. Ç., Kara, İ & Yıkılmaz, S. K. Quantification of the forearm muscles mechanical properties using Myotonometer: Intra- and inter-examiner reliability and its relation with hand grip strength. *J. Electromyogr. Kinesiol.* **67**, 102718 (2022). 36334405 10.1016/j.jelekin.2022.102718

[39] Koch, V. & Wilke, J. Reliability of a new indentometer device for measuring myofascial tissue stiffness. *J. Clin. Med.* **11**, 5194 (2022). 36079124 10.3390/jcm11175194 PMC9457058

[40] Wernicke, A. G. et al. Tissue Compliance Meter is a more reproducible method of measuring radiation-induced fibrosis than Late Effects of Normal Tissue-Subjective Objective Management Analytical in patients treated with intracavitary brachytherapy accelerated partial breast irradiation: results of a prospective trial. *Breast J.* **19**, 250–258 (2013). 23614363 10.1111/tbj.12102

[41] Wilke, J. et al. Reliability and validity of a semi-electronic tissue compliance meter to assess muscle stiffness. *J. Back Musculoskelet. Rehabil.* **31**, 991–997 (2018). 29945340 10.3233/BMR-170871

[42] Bartsch, K. et al. Assessing reliability and validity of different stiffness measurement tools on a multi-layered phantom tissue model. *Sci. Rep.* **13**, 815 (2023). 36646734 10.1038/s41598-023-27742-w PMC9842673

[43] Arnold, G. et al. Normal magnetic resonance imaging anatomy of the ankle & foot. *Magn. Reson. Imaging Clin. N. Am.* **19**, 655–679 (2011). 21816337 10.1016/j.mric.2011.05.010

[44] Maemichi, T. et al. Changes in functional characteristics of heel fat pad with age. *Clin. Biomech.* **118**, 106294 (2024). 10.1016/j.clinbiomech.2024.106294 38996494

[45] Malo-Urriés, M. et al. The precision and safety of ultrasound-guided versus palpation-guided needle placement on the plantar fascia and flexor digitorum brevis interface: an anatomical study. *Healthcare* **12**, 1000 (2024). 38786411 10.3390/healthcare12101000 PMC11121310

[46] Mix, A. & Giacomin, A. Standardized polymer durometry. *J. Test. Eval.* **39**, 696–705 (2011).

[47] Ryu, J. & Jeong, W. K. Current status of musculoskeletal application of shear wave elastography. *Ultrasonography* **36**, 185–193 (2017). 28292005 10.14366/usg.16053 PMC5494870

[48] O’Hara, S., Edwards, C. & Zelesco, M. Two dimensional shear wave elastography—Basic principles and current applications. *Sonography* **11**, 201–210 (2024).

[49] Brandl, A. et al. Reliability and validity of an app-assisted tissue compliance meter in measuring tissue stiffness on a phantom model. In: *Presented at Scientific Conference* (2023). 10.7717/peerj.17122 PMC10924762 38464760

[50] Ghosh, S. et al. mTG-Gelatin phantoms as standardized testbeds for skin biomechanical measurements with Myoton. *J. Mech. Behav. Biomed. Mater.* **158**, 106651 (2024). 39059120 10.1016/j.jmbbm.2024.106651 PMC11908466

[51] Falanga, V. & Bucalo, B. Use of a durometer to assess skin hardness. *J. Am. Acad. Dermatol.* **29**, 47–51 (1993). 8315077 10.1016/0190-9622(93)70150-r

[52] Periyasamy, R., Anand, S. & Ammini, A. Investigation of Shore meter in assessing foot sole hardness in patients with diabetes mellitus—A pilot study. *Int. J. Diabetes Dev. Ctries.* **32**, 169–175 (2012).

[53] Fischer, A. Tissue compliance meter for objective, quantitative documentation of soft tissue consistency and pathology. *Arch. Phys. Med. Rehabil.* **68**, 122–125 (1987). 3813858

[54] Brandl, A. et al. Reliability and validity of an app-assisted tissue compliance meter in measuring tissue stiffness on a phantom model. *PeerJ* **12**, e17122 (2024). 38464760 10.7717/peerj.17122 PMC10924762

[55] R Core Team. R: A language and environment for statistical computing (Version 4.3.1) [Computer software] (R Foundation for Statistical Computing, 2023).

[56] Bland, J. M. & Altman, D. Statistical methods for assessing agreement between two methods of clinical measurement. *Lancet* **327**, 307–310 (1986). 2868172

[57] Cohen, J. *Statistical Power Analysis for the Behavioral Sciences* (Routledge, 2013).

[58] Bakeman, R. Recommended effect size statistics for repeated measures designs. *Behav. Res. Methods* **37**, 379–384 (2005). 16405133 10.3758/bf03192707

[59] Nguyen, A. P. et al. Myotonpro is a valid device for assessing wrist biomechanical stiffness in healthy young adults. *Front. Sports Act. Living* **4**, 797975 (2022). 35265831 10.3389/fspor.2022.797975 PMC8899712

[60] Shan, X. et al. Biomechanical assessment of gastrocnemii and Achilles tendon using MyotonPRO: In vivo measurements, and preliminary in situ measurements using formalin-fixed tissues. *Connect. Tissue Res.* **65**, 16–25 (2024). 37830341 10.1080/03008207.2023.2267682

[61] Bouffandeau, A. et al. Assessment of the mechanical properties of soft tissue phantoms using impact analysis. *Sensors* **25**, 1344 (2025). 40096164 10.3390/s25051344 PMC11902684

[62] Romero, S. E. et al. Plantar soft tissue characterization using reverberant shear wave elastography: A proof-of-concept study. *Ultrasound Med. Biol.* **48**, 35–46 (2022). 34702642 10.1016/j.ultrasmedbio.2021.09.011

[63] Dalal, S., Widgerow, A. D. & Evans, G. R. The plantar fat pad and the diabetic foot—A review. *Int. Wound J.* **12**, 636–640 (2015). 24131727 10.1111/iwj.12173 PMC7950511

[64] Bai, X. et al. Development of an interpretable model for foot soft tissue stiffness based on gait plantar pressure analysis. *Front. Bioeng. Biotechnol.* **12**, 1482382 (2025). 39834637 10.3389/fbioe.2024.1482382 PMC11743706

[65] Orner, S. et al. Quantitative tissue parameters of Achilles tendon and plantar fascia in healthy subjects using a handheld myotonometer. *J. Bodyw. Mov. Ther.* **22**, 105–111 (2018). 29332731 10.1016/j.jbmt.2017.06.015

[66] Albano, D. et al. Shear-wave elastography of the plantar fascia: A systematic review and meta-analysis. *J. Ultrasound* **26**, 59–64 (2023). 36662404 10.1007/s40477-022-00770-4 PMC10063692

[67] Gatz, M. et al. Shear wave elastography (SWE) for the evaluation of patients with plantar fasciitis. *Acad. Radiol.* **27**, 363–370 (2020). 31153782 10.1016/j.acra.2019.04.009

[68] Jiao, X. et al. Association between elastic modulus of foot soft tissues and gait characteristics in young individuals with flatfoot. *Bioengineering* **11**, 728 (2024). 39061810 10.3390/bioengineering11070728 PMC11273929

[69] Schillizzi, G. et al. Evaluation of plantar fasciopathy shear wave elastography: A comparison between patients and healthy subjects. *J. Ultrasound* **24**, 417–422 (2021). 32418168 10.1007/s40477-020-00474-7 PMC8572281

[70] Wang, K. et al. Noninvasive in vivo study of the morphology and mechanical properties of plantar fascia based on ultrasound. *IEEE Access* **7**, 53641–53649 (2019).

[71] Costello, C. et al. The importance of preconditioning for the sonographic assessment of plantar fascia thickness and shear wave velocity. *Sensors* **24**, 4552 (2024). 39065950 10.3390/s24144552 PMC11280931

[72] Chatzistergos, P. E. et al. Shear wave elastography can assess the in-vivo nonlinear mechanical behavior of heel-pad. *J. Biomech.* **80**, 144–150 (2018). 30241799 10.1016/j.jbiomech.2018.09.003

