# Supplementary material for: Validation and reliability of mechanical stiffness assessment tools in multilayered polyurethane phantom models of healthy and diabetic plantar soft tissues
Source: Sci Rep. 2025 Sep 30;15:34118. doi: 10.1038/s41598-025-21084-5 (PMC12484676; doi:10.1038/s41598-025-21084-5)
Supplement: Supplementary file 1 — Supplementary Material 1 [file 41598_2025_21084_MOESM1_ESM.docx]

**Supplemental File 1:** Literature-Based Reference Data for Plantar Tissue Properties, categorized as follows: Plantar skin, plantar fat pad, plantar fascia, and ıntrinsic foot muscles.

**Plantar Skin Thickness and Stiffness Values**

| **Region** | Healthy Thickness (mm) | Diabetic Thickness (mm) | Healthy SH Stiffness | Diabetic SH Stiffness |
| --- | --- | --- | --- | --- |
| **Calcaneal** | 1.70 [1]  1.32 [2]  1.8 [3] | 2.056 [3] | 30 Shore OO(SH) (= 105.35 kPa (20 to 41 = 69.76 to 164.74 kPa) [4]  56 SH= 316.35 kPa [2]  20 SH= 69.76 kPa [5]  44-51 SH= 186.59-252.01 kPa [6] | 62+ SH = 424.33 kPa [2]  30-40 SH = 105.35 kPa to 158.10 kPa [5] |
| **Mid foot** | 0.8, min: 0.6, max: 1.2 [4]  0.7 ± 0.2 [7] | 0.6 - 1.1 [4]  0.4 ± 0.1 [7] | 39-43 SH= 151.76 -178.96 kPa [4] | 49-51 SH= 230.86 – 252.01 kPa [4] |
| **Forefoot (MTHs)** | 2.4 - 2.6 [8] | 1.7 - 1.9 [8] | 44 SH= 186.59 kPa [4] | 51 SH = 252.01 kPa [4] |

**Plantar Fat Pad Thickness and Stiffness Values**

| **Region** | **Healthy Thickness (mm)** | **Diabetic Thickness (mm)** | | **Healthy SH Stiffness** | **Diabetic SH Stiffness** |
| --- | --- | --- | --- | --- | --- |
| **Calcaneal**  **Heel Pad** | 19.5 ± 4.7 [9]  18.4 ± 12.0 [10]  15.5 ± 2.4 [11]  16.5 ± 1.9 [12]  18.62 (SE 0.36) [13]  16.6 (SEM 0.32) [13]  14.6 ± 3.1 [5]  18.87 ± 0.65 [14]  10.8 ± 1.6 [15]  13.17±1.68 [16] | 19.4 ± 3.5 [9]  19.3 ± 3.0 [10]  16.1 ± 2.4 [11]  17.2 ± 3.1 [12]  17.33 (SE 0.29) [13]  17.8 (SEM 0.31) [13]  14.3 ± 1.5 [5]  18.25 ± 0.52 [14]  10.3 ± 0.9 [15]  14.98 ±1.76 [16] | | 65.62 ± 4.10 kPa  [14] (34 SH)  168 ±30 kPa [12] (47 SH)  23.2±4.1, 23.5±4.5, and 23.7±4.0 kPa / (20 SH) [17]  221 (SD 45) kPa / (51 SH) [10]  175 kPa / (48 SH) [18]  192.55 and 197.585 kPa (49 SH – 50 SH) [19] | 44.95 ± 4.20 kPa ( 29.75 SH)  [14]  161 ± 19 kPa (46.92 ± 18.6 SH)  170 ±32 kPa (47.65 ± 25.17 SH) [12]  241 ± 54 kPa (52.35 ±32.22 SH) [10]  6.60 (± 4.41) equal to 149.16 ± 99.67 kPa  (45.89 ±40.46 SH) [20]  2.71 to 2.27 MPa / 4.68 ±0.87MPa [21] |
| **Mid foot** | 6.0 ± 1.2 [22]  It ranges between 7.5 and 8 mm in individuals aged 20 to 69 years. | sub-MTH fat-pad thickness  2.5 ± 1.3 vs. 6.0 ± 1.4 [22] | Between the ages of 20 and 69, the shear modulus ranges from 11.5 to 15.0 kPa, which corresponds to a Young's modulus of approximately 29.90 to 39.0 kPa. [15] (24 SH- 28 SH)  Bulk Heel Pad: 31.8±6.3 to 34.2±6.6 kPa  (25 SH-26 SH) [23] | | Diseased Heel Pad 46.6±10.9 kPa  (30.23 ± 10,68 SH) [23] |
| **Forefoot** | 4.9 to 6.5 [8]  7.67 to 8.74 [14]  7.7 ± 1.3 [22] | 3.9 to 5.2 [8]  8.25 to 8.88 [14]  subphalangeal fat-pad thickness  7.6 ± 1.2 to 9.1 ± 1.9 [22] | 84.77 to 96.29 kPa [14]  (38 SH to 40 SH)  According to reported values, the shear modulus in individuals aged 20 to 69 years ranges from 10.7 to 15.2 kPa, corresponding to a Young’s modulus of 27.82 to 39.52 kPa. (23 SH to 28 SH) [15] | | 10.61 (± 7.27) equal to 239.79 ± 164.30 (75.49 to 404.09 kPa = 36.72 to 59.30 SH) [20, 24] |

**Plantar Fascia Thickness and Stiffness Values**

| **Region** | **Healthy Thickness (mm)** | **Diabetic Thickness (mm)** | **Healthy SH Stiffness** | | **Diabetic SH Stiffness** |
| --- | --- | --- | --- | --- | --- |
| **Calcaneal** | 2.79 to 3.90 [25] 2.0 ± 0.5 [26] 3.3 [27] 3.06 to 2.83 [28] 4.41 ±1.0 [29]  3.035 ± 0.53 [30] 2.68 ± 0.59 [31] 3.0 (5.1- 2.5) [32]  3.53 ± 0.8 [16] 3.0 ± 0.6 [33] | D: 2.9 ± 1.2 [26]  DN: 3.0 ± 0.8 [26]  3.74 ± 0.76 [34]  4.20 ± 0.85 [34]  3.65 ± 0.88 [31]  3.89 ± 0.92 [16] 3.7 ± 0.9 [33] | 130.0 ± 47.0 kPa (44.04±30.35 SH)  168 to 304 kPa (47.49 to 55.47 SH) [25]  SWE 5.4 (0.6) m/s= 87.48 kPa (38.71 SH) (69.12–108.00 kPa= 35.54- 41.54 SH) [35]  146.9 kPa (45.69 SH) [29] 152.88 kPa (46.22 SH) [36] | | 161.0 ±94.1 kPa  (~255 kPa=~53 SH) (46.92±39.69 SH) [16]  129.4 kPa in the group with injuries (43.98 SH) [29] |
| **Mid foot** | 2.0 ± 0.5 [37]  (3.1±0.4 (2.5-3.8) [38]  1.76 ± 0.32 [39] 2.50 ± 0.50 [39] 2.11 ± 0.41 [39]  2.8 ± 0.3 [40] 2.47 to 2.26 [28]  2.6±0.4 [8]  1.7 [32]  1.87±0.33 [16]  1.97 ± 0.19 [41] | D: 2.9 ± 1.2 [37]  DN: 3.0 ± 0.8 [37]  DNPU: 3.1 ± 1.0 [37]  3.5 ± 0.7 (BMI < 25), [40]  4.3 ± 1.3 ( BMI 25–30) [40]  5.3 ± 0.7 (BMI > 30) [40]  3.5±0.4 [8]  1.90± 0.40 [16] 2.53 ± 0.51 [41] | 6.5 m/s is 126.75 kPa. (43.70 SH) [32]  6.94 m/s = 144.49 kPa (45.46 SH) [36]  Cut-off: SWE velocity 6.16 m/s, stiffness 125.57 kPa) (43.57. SH) [36]  6.28±0.88 m/s = 118.32 kPa (42.77 SH) (87.48–153.80 kPa= 38.71- 46.30 SH) [41]  64.7±9.4 kPa (34.65±8.69 SH)  Young healthy subjects [42]  PF (93.3 kPa, (39.58 SH) 5.58 m/s) [43]  72.0 ±37.0 kPa (36.09± 27.13 SH) [16] | Pathologic PFs had lower SWE velocity (4.98 m/s= 74.40 kPa) (36.53 SH) [36]  4.53±0.89 m/s = 61.56 kPa (39.75–88.13 kPa) 32.87 SH (28.09-38.81 SH) [41]  Cut-off point for positive SWE finding was 51.5 kPa (31.58 SH) / (4.14 m/s). Symptomatic PF (31.9 kPa, 3.26 m/s) (25.13 SH) [43] | |
| **Forefoot (MTHs)** | 1st MTs 1.26 to 1.64  2. MTS 0.90 to 1.39  3. MTs 0.98 to 1.35  4. MTs 0.70 to 1.15  5. MTS 0.40 to 1.00 [25] 1.5 (1.9 to 0.8) [32] 1.4±0.3 [16] | 1.44 ± 0.4 [16] | 40 to 61.38 kPa (28.18 to 33.94 SH)  27.6 to 37.5 kPa (23.18 to 27.31 SH) 27.0 to 43.08 kPa (22.89 to 29.18 SH) 17.0 to 28.90 kPa (16.66 to 23.80 SH)  14.7 to 25.93 kPa (14.70 to 22.34 SH) [25] | | 97.2 ±56.7 kPa  40.5 - 153.9  (28.34 – 46.31 SH) [16] |

**Intrinsic Foot MusclesThickness and Stiffness Values**

| **Muscle** | Healthy Thickness (mm) | Diabetic Thickness (mm) | | Healthy SH Stiffness (kPa) | Diabetic SH Stiffness (kPa) | |
| --- | --- | --- | --- | --- | --- | --- |
| **Abductor Hallucis** | 10.5 ± 2.1 [38]  10.8-12.2 [44]  11.80 ± 0.39 [16] 12.00 [45] | 11.10 [46]  11.90 ± 2.0 [16] | 31.1 ± 11.4 kPa (24,79 ±11,28 SH) 25.11 ± -10.04 kPa (21.91±9.57 SH) 33.73 (45.2−24.04) kPa (25.88 (29.82-21.32 SH) [45] 10.1 ± 2.1 kPa (9,65 SH) [47] | | 27.7 ± 9.5 kPa  (23.23 ± 8.83 SH)  (18.2 to 37.2 kpa)  (17.58 to 27.20 SH) [16] | |
| **Flexor Digitorum Brevis** | 9.0 ± 1.4 [38]  10.2-10.4 [44]  9.50 ± 1.6 [48]  9.90 (7.8 – 11.1) [45] | 8.70 [44] | 13.0 ± 1.8 kPa (13.05 SH) [48]  8.07 ± -1.42 kPa (6.63 SH)  8.79 (14.12−5.79) kPa [7.78 kPa (14.16 – 2.16 SH) [45]  8.1 ± 1.6 kPa (6.68 SH) [47] | | | - |

**Supplemental File 2.** Coding Scheme and Configuration Matrix of Multilayer Phantom Models Across Anatomical Regions

| **Healthy Model: Calcaneal Region** | | | **Healthy Model: Midfoot Region** | | | | **Healthy Model: Forefoot Region** | | | |
| --- | --- | --- | --- | --- | --- | --- | --- | --- | --- | --- |
| **Layer 1** | **Layer 2** | **Layer 3** | **Layer 1** | **Layer 2** | **Layer 3** | **Layer 4 (Fixed)** | **Layer 1 (Fixed)** | **Layer 2** | **Layer 3** | **Layer 4** |
| A48742 | A21083 | A11179 | A37718 | B90057 | B32372 | B72788 | A11108 | A39415 | A46742 | A21917 |
| A48742 | A21083 | B54659 | A37718 | B90057 | B54659 | B72788 | A11108 | A39415 | A46742 | A21083 |
| A48742 | A21083 | B64504 | A37718 | B90057 | B64504 | B72788 | A11108 | A39415 | A46742 | A86691 |
| A48742 | A36907 | A11179 | A37718 | B30216 | B32372 | B72788 | A11108 | A39415 | A26181 | A21917 |
| A48742 | A36907 | B54659 | A37718 | B30216 | B54659 | B72788 | A11108 | A39415 | A26181 | A21083 |
| A48742 | A36907 | B64504 | A37718 | B30216 | B64504 | B72788 | A11108 | A39415 | A26181 | A86691 |
| A48742 | A88020 | A11179 | A37718 | B22332 | B32372 | B72788 | A11108 | A39415 | A36718 | A21917 |
| A48742 | A88020 | B54659 | A37718 | B22332 | B54659 | B72788 | A11108 | A39415 | A36718 | A21083 |
| A48742 | A88020 | B64504 | A37718 | B22332 | B64504 | B72788 | A11108 | A39415 | A36718 | A86691 |
| B66583 | A21083 | A11179 | A45327 | B90057 | B32372 | B72788 | A11108 | A15481 | A46742 | A21917 |
| B66583 | A21083 | B54659 | A45327 | B90057 | B54659 | B72788 | A11108 | A15481 | A46742 | A21083 |
| B66583 | A21083 | B64504 | A45327 | B90057 | B64504 | B72788 | A11108 | A15481 | A46742 | A86691 |
| B66583 | A34907 | A11179 | A45327 | B30216 | B32372 | B72788 | A11108 | A15481 | A26181 | A21917 |
| B66583 | A34907 | B54659 | A45327 | B30216 | B54659 | B72788 | A11108 | A15481 | A26181 | A21083 |
| B66583 | A34907 | B64504 | A45327 | B30216 | B64504 | B72788 | A11108 | A15481 | A26181 | A86691 |
| B66583 | A88020 | A11179 | A45327 | B22332 | B32372 | B72788 | A11108 | A15481 | A36718 | A21917 |
| B66583 | A88020 | B54659 | A45327 | B22332 | B54659 | B72788 | A11108 | A15481 | A36718 | A21083 |
| B66583 | A88020 | B64504 | A45327 | B22332 | B64504 | B72788 | A11108 | A15481 | A36718 | A86691 |
| A46490 | A21083 | A11179 | A98575 | B90057 | B32372 | B72788 | A11108 | A91825 | A46742 | A21917 |
| A46490 | A21083 | B54659 | A98575 | B90057 | B54659 | B72788 | A11108 | A91825 | A46742 | A21083 |
| A46490 | A21083 | B64504 | A98575 | B90057 | B64504 | B72788 | A11108 | A91825 | A46742 | A86691 |
| A46490 | A34907 | A11179 | A98575 | B30216 | B32372 | B72788 | A11108 | A91825 | A26181 | A21917 |
| A46490 | A34907 | B54659 | A98575 | B30216 | B54659 | B72788 | A11108 | A91825 | A26181 | A21083 |
| A46490 | A34907 | B64504 | A98575 | B30216 | B64504 | B72788 | A11108 | A91825 | A26181 | A86691 |
| A46490 | A88020 | A11179 | A98575 | B22332 | B32372 | B72788 | A11108 | A91825 | A36718 | A21917 |
| A46490 | A88020 | B54659 | A98575 | B22332 | B54659 | B72788 | A11108 | A91825 | A36718 | A21083 |
| A46490 | A88020 | B64504 | A98575 | B22332 | B64504 | B72788 | A11108 | A91825 | A36718 | A86691 |
| **Diabetic Model: Calcaneal Region** | | | **Diabetic Model: Midfoot Region** | | | | **Diabetic Model: Forefoot Region** | | | |
| **Layer 1** | **Layer 2** | **Layer 3** | **Layer 1** | **Layer 2** | **Layer 3** | **Layer 4 (Fixed)** | **Layer 1 (Fixed)** | **Layer 2** | **Layer 3** | **Layer 4** |
| A66578 | A35907 | A62934 | B10465 | B37649 | A48453 | B67931 | A96785 | A94024 | A93544 | B72788 |
| A66578 | A35907 | A97785 | B10465 | B37649 | A13937 | B67931 | A96785 | A94024 | A93544 | A86691 |
| A66578 | A35907 | A67641 | B10465 | B37649 | A31618 | B67931 | A96785 | A94024 | A93544 | A36907 |
| A66578 | A90275 | A62934 | B10465 | B17501 | A48453 | B67931 | A96785 | A94024 | B66583 | B72788 |
| A66578 | A90275 | A97785 | B10465 | B17501 | A13937 | B67931 | A96785 | A94024 | B66583 | A86691 |
| A66578 | A90275 | A67641 | B10465 | B17501 | A31618 | B67931 | A96785 | A94024 | B66583 | A36907 |
| A66578 | A88020 | A62934 | B10465 | B35465 | A48453 | B67931 | A96785 | A94024 | A98575 | B72788 |
| A66578 | A88020 | A97785 | B10465 | B35465 | A13937 | B67931 | A96785 | A94024 | A98575 | A86691 |
| A66578 | A88020 | A67641 | B10465 | B35465 | A31618 | B67931 | A96785 | A94024 | A98575 | A36907 |
| A62934 | A35907 | A62934 | B42029 | B37649 | A48453 | B67931 | A96785 | A90361 | A93544 | B72788 |
| A62934 | A35907 | A97785 | B42029 | B37649 | A13937 | B67931 | A96785 | A90361 | A93544 | A86691 |
| A62934 | A35907 | A67641 | B42029 | B37649 | A31618 | B67931 | A96785 | A90361 | A93544 | A36907 |
| A62934 | A90275 | A62934 | B42029 | B17501 | A48453 | B67931 | A96785 | A90361 | B66583 | B72788 |
| A62934 | A90275 | A97785 | B42029 | B17501 | A13937 | B67931 | A96785 | A90361 | B66583 | A86691 |
| A62934 | A90275 | A67641 | B42029 | B17501 | A31618 | B67931 | A96785 | A90361 | B66583 | A36907 |
| A62934 | A88020 | A62934 | B42029 | B35465 | A48453 | B67931 | A96785 | A90361 | A98575 | B72788 |
| A62934 | A88020 | A97785 | B42029 | B35465 | A13937 | B67931 | A96785 | A90361 | A98575 | A86691 |
| A62934 | A88020 | A67641 | B42029 | B35465 | A31618 | B67931 | A96785 | A90361 | A98575 | A36907 |
| A19599 | A35907 | A62934 | B77153 | B37649 | A48453 | B67931 | A96785 | A73645 | A93544 | B72788 |
| A19599 | A35907 | A97785 | B77153 | B37649 | A13937 | B67931 | A96785 | A73645 | A93544 | A86691 |
| A19599 | A35907 | A67641 | B77153 | B37649 | A31618 | B67931 | A96785 | A73645 | A93544 | A36907 |
| A19599 | A90275 | A62934 | B77153 | B17501 | A48453 | B67931 | A96785 | A73645 | B66583 | B72788 |
| A19599 | A90275 | A97785 | B77153 | B17501 | A13937 | B67931 | A96785 | A73645 | B66583 | A86691 |
| A19599 | A90275 | A67641 | B77153 | B17501 | A31618 | B67931 | A96785 | A73645 | B66583 | A36907 |
| A19599 | A88020 | A62934 | B77153 | B35465 | A48453 | B67931 | A96785 | A73645 | A98575 | B72788 |
| A19599 | A88020 | A97785 | B77153 | B35465 | A13937 | B67931 | A96785 | A73645 | A98575 | A86691 |
| A19599 | A88020 | A67641 | B77153 | B35465 | A31618 | B67931 | A96785 | A73645 | A98575 | A36907 |

**References**

1. Brady L, et al. The compressive, shear, biochemical, and histological characteristics of diabetic and non-diabetic plantar skin are minimally different. *Journal of Biomechanics* 129, 110797 (2021).
2. Chatzistergos PE, et al. Shore hardness is a more representative measurement of bulk tissue biomechanics than of skin biomechanics. *Medical Engineering & Physics* 105, 103816 (2022).
3. Wang YN, Lee K, Ledoux WR. Histomorphological evaluation of diabetic and non-diabetic plantar soft tissue. *Foot & Ankle International* 32, 802–810 (2011).
4. Tonna R, et al. Reliability and validity of shore hardness in plantar soft tissue biomechanics. *Sensors* 24, 539 (2024).
5. Thomas VJ, et al. The role of skin hardness, thickness, and sensory loss on standing foot power in the development of plantar ulcers in patients with diabetes mellitus—a preliminary study. *The International Journal of Lower Extremity Wounds* 2, 132–139 (2003).
6. Piaggesi A, et al. Hardness of plantar skin in diabetic neuropathic feet. *Journal of Diabetes Complications* 13, 129–134 (1999).
7. Petrofsky JS, Prowse M, Lohman E. The influence of ageing and diabetes on skin and subcutaneous fat thickness in different regions of the body. *Journal of Applied Research* 8, (2008).
8. Kumar CGS, et al. Intrinsic foot muscle and plantar tissue changes in type 2 diabetes mellitus. *Journal of Diabetes* 7, 850–857 (2015).
9. Chatzistergos PE, et al. The relationship between the mechanical properties of heel-pad and common clinical measures associated with foot ulcers in patients with diabetes. *Journal of Diabetes and its Complications* 28, 488–493 (2014).
10. Hsu CC, et al. Diabetic effects on microchambers and macrochambers tissue properties in human heel pads. *Clinical Biomechanics* 24, 682–686 (2009).
11. Tong J, Lim C, Goh O. Technique to study the biomechanical properties of the human calcaneal heel pad. *The Foot* 13, 83–91 (2003).
12. Hsu TC, et al. Altered heel‐pad mechanical properties in patients with type 2 diabetes mellitus. *Diabetic Medicine* 17, 854–859 (2000).
13. Gooding GA, et al. Sonography of the sole of the foot: evidence for loss of foot pad thickness in diabetes and its relationship to ulceration of the foot. *Investigative Radiology* 21, 45–48 (1986).
14. Sun JH, et al. Changes in the thickness and stiffness of plantar soft tissues in people with diabetic peripheral neuropathy. *Archives of Physical Medicine and Rehabilitation* 92, 1484–1489 (2011).
15. Mo F, et al. In vivo measurement of plantar tissue characteristics and its indication for foot modeling. *Annals of Biomedical Engineering* 47, 2356–2371 (2019).
16. Bell EA. Stiffness of intrinsic foot structures in diabetic individuals and the effect of stiffness on plantar pressures during gait. MSc thesis, East Carolina University (2019).
17. Lin CY, et al. Spatial-dependent mechanical properties of the heel pad by shear wave elastography. *Journal of Biomechanics* 53, 191–195 (2017).
18. Gefen A. Plantar soft tissue loading under the medial metatarsals in the standing diabetic foot. *Medical Engineering & Physics* 25, 491–499 (2003).
19. Teng ZL, et al. Effect of loading history on material properties of human heel pad: an in-vivo pilot investigation during gait. *BMC Musculoskeletal Disorders* 23, 254 (2022).
20. Naemi R, et al. Can plantar soft tissue mechanics enhance prognosis of diabetic foot ulcer? *Diabetes Research and Clinical Practice* 126, 182–191 (2017).
21. Kwak Y, et al. Increase of stiffness in plantar fat tissue in diabetic patients. *Journal of Biomechanics* 107, 109857 (2020).
22. Bus SA, Akkerman EM, Maas M. Changes in sub-calcaneal fat pad composition and their association with dynamic plantar foot pressure in people with diabetic neuropathy. *Clinical Biomechanics* 88, 105441 (2021).
23. Lin CY, et al. Heel pad stiffness in plantar heel pain by shear wave elastography. *Ultrasound in Medicine & Biology* 41, 2890–2898 (2015).
24. Naemi R, et al. Diabetes status is associated with plantar soft tissue stiffness measured using ultrasound reverberant shear wave elastography approach. *Journal of Diabetes Science and Technology* 16, 478–490 (2022).
25. Wang K, et al. Noninvasive in vivo study of the morphology and mechanical properties of plantar fascia based on ultrasound. *IEEE Access* 7, 53641–53649 (2019).
26. Giacomozzi C, et al. Does the thickening of Achilles tendon and plantar fascia contribute to the alteration of diabetic foot loading? *Clinical Biomechanics* 20, 532–539 (2005).
27. Gibbon W, Long G. Ultrasound of the plantar aponeurosis (fascia). *Skeletal Radiology* 28, 21–26 (1999).
28. Bisi-Balogun A, Cassel M, Mayer F. Reliability of various measurement stations for determining plantar fascia thickness and echogenicity. *Diagnostics* 6, 15 (2016).
29. Aguilar-Nuñez D, et al. Ultrasound strain elastography reliability in the assessment of the plantar fascia and its relationship with the plantar thickness in healthy adults: an intra and interobserver reliability study in novice evaluators. *Biomedicines* 11, 2040 (2023).
30. Abul K, et al. Detection of normal plantar fascia thickness in adults via the ultrasonographic method. *Journal of the American Podiatric Medical Association* 105, 8–13 (2015).
31. Dixit R, Singh S, Garg S. Evaluation of the plantar fascia in patients with diabetes mellitus: the role of sonoelastography. *Polish Journal of Radiology* 87, 500–505 (2022).
32. Costello C, et al. The importance of preconditioning for the sonographic assessment of plantar fascia thickness and shear wave velocity. *Sensors* 24, 4552 (2024).
33. Ursini F, et al. Plantar fascia enthesopathy is highly prevalent in diabetic patients without peripheral neuropathy and correlates with retinopathy and impaired kidney function. *PLoS One* 12, e0174529 (2017).
34. Santoboni F, et al. Correlates of the ultrasonographic and elastosonographic parameters of the plantar fascia in patients with type 2 diabetes. *Muscles, Ligaments & Tendons Journal* 13, (2023).
35. Chino K, et al. Effect of toe dorsiflexion on the regional distribution of plantar fascia shear wave velocity. *Clinical Biomechanics* 61, 11–15 (2019).
36. Baur D, et al. Shear wave elastography of the plantar fascia: comparison between patients with plantar fasciitis and healthy control subjects. *Journal of Clinical Medicine* 10, 2351 (2021).
37. D’ambrogi E, et al. Abnormal foot function in diabetic patients: the altered onset of Windlass mechanism. *Diabetic Medicine* 22, 1713–1719 (2005).
38. Taş S, Çetin A. An investigation of the relationship between plantar pressure distribution and the morphologic and mechanic properties of the intrinsic foot muscles and plantar fascia. *Gait & Posture* 72, 217–221 (2019).
39. Jha DK, Wongkaewpotong J, Chuckpaiwong B. Effect of age and BMI on sonographic findings of plantar fascia. *The Journal of Foot and Ankle Surgery* 62, 125–128 (2023).
40. Abate M, et al. Achilles tendon and plantar fascia in recently diagnosed type II diabetes: role of body mass index. *Clinical Rheumatology* 31, 1109–1113 (2012).
41. Saroha A, et al. Ultrasonographic evaluation of thickness and stiffness of Achilles tendon and plantar fascia in type 2 diabetics patients: a cross-sectional observation study. *Journal of Medical Ultrasound* 31, 282–286 (2023).
42. Nozaki S, et al. Quantification of the in vivo stiffness and natural length of the human plantar aponeurosis during quiet standing using ultrasound elastography. *Scientific Reports* 12, 15707 (2022).
43. Gatz M, et al. Shear wave elastography (SWE) for the evaluation of patients with plantar fasciitis. *Academic Radiology* 27, 363–370 (2020).
44. Haelewijn N, et al. Quantitative ultrasonography of the foot muscles: a comprehensive perspective on reliability. *Quantitative Imaging in Medicine and Surgery* 15, 203 (2024).
45. Maeda N, et al. Intrinsic foot muscle hardness is related to dynamic postural stability after landing in healthy young men. *Gait & Posture* 86, 192–198 (2021).
46. Haelewijn N, et al. Current evidence regarding 2D ultrasonography monitoring of intrinsic foot muscle properties: a systematic review. *Heliyon* 9, (2023).
47. Hirota K, et al. Comparison by ultrasound shear wave elastography of toe flexor muscle contraction during MTP flexion exercise and short-foot exercise. *Journal of Back and Musculoskeletal Rehabilitation* 37, 1041–1047 (2024).
48. Jiao X, et al. Association between elastic modulus of foot soft tissues and gait characteristics in young individuals with flatfoot. *Bioengineering* 11, 728 (2024).
